# Flipping chromosomes in deep-sea archaea

**DOI:** 10.1371/journal.pgen.1006847

**Published:** 2017-06-19

**Authors:** Matteo Cossu, Catherine Badel, Ryan Catchpole, Danièle Gadelle, Evelyne Marguet, Valérie Barbe, Patrick Forterre, Jacques Oberto

**Affiliations:** 1 Institute for Integrative Biology of the Cell (I2BC), Microbiology Department, CEA, CNRS, Univ. Paris‐Sud, Université Paris‐Saclay, Gif‐sur‐Yvette, France; 2 Genoscope, Laboratoire de Biologie Moléculaire pour l'Etude des Génomes C.E.A., Institut de Génomique - 2 rue Gaston Crémieux, EVRY, France; Max Planck Institute for Terrestrial Microbiology, GERMANY

## Abstract

One of the major mechanisms driving the evolution of all organisms is genomic rearrangement. In hyperthermophilic Archaea of the order *Thermococcales*, large chromosomal inversions occur so frequently that even closely related genomes are difficult to align. Clearly not resulting from the native homologous recombination machinery, the causative agent of these inversions has remained elusive. We present a model in which genomic inversions are catalyzed by the integrase enzyme encoded by a family of mobile genetic elements. We characterized the integrase from *Thermococcus nautili* plasmid pTN3 and showed that besides canonical site-specific reactions, it catalyzes low sequence specificity recombination reactions with the same outcome as homologous recombination events on DNA segments as short as 104bp both *in vitro* and *in vivo*, in contrast to other known tyrosine recombinases. Through serial culturing, we showed that the integrase-mediated divergence of *T*. *nautili* strains occurs at an astonishing rate, with at least four large-scale genomic inversions appearing within 60 generations. Our results and the ubiquitous distribution of pTN3-like integrated elements suggest that a major mechanism of evolution of an entire order of Archaea results from the activity of a selfish mobile genetic element.

## Author summary

Mobile elements (MEs) such as viruses, plasmids and transposons infect most living organisms and often encode recombinases promoting their insertion into cellular genomes. These insertions alter the genome of their host according to two main mechanisms. First, MEs provide new functions to the cell by integrating their own genetic information into the DNA of the host, at one or more locations. Secondly, cellular homologous recombination will act upon multiple integrated copies and produce a variety of large-scale chromosomal rearrangements. If such modifications are advantageous, they will spread into the population by natural selection. Typically, enzymes involved in cellular homologous recombination and the integration of MEs are distinct. We describe here a novel plasmid-encoded archaeal integrase which in addition to site-specific recombination can catalyze low sequence specificity recombination reactions akin to homologous recombination.

## Introduction

Large-scale genomic rearrangements allow organisms to evolve much more rapidly than through random mutation alone. Rearrangements can result in the movement of genes within genomes, changes in coding strand use, loss of nonessential functions and the incorporation of foreign DNA. As a result, the organization, content and processing of genetic information can be deeply altered. In all three domains of life, chromosomal reorganization is mainly promoted by recombination between homologous sequences, for example between redundant ribosomal operons [1,2] or integrated copies of mobile elements (ME) such as prophages [3,4], transposons [5,6] and insertion sequences (IS) [7]. Such recombination can result in the DNA inversions readily observed in closely related genomes [8,9]. In addition to homologous recombination, chromosomes can undergo rearrangement through retrotransposon-associated non-homologous recombination [10]. Other elements like integrons confer rapid adaptation to bacteria in changing environments by shuffling cassette arrays encoding a variety of functions, a process involving a site-specific recombinase and two types of attachment sites [11]. Further genomic rearrangement/reorganization can occur through the acquisition of new genetic material, predominantly by lateral gene transfer. Such gene transfer occurs in all organisms through infection by mobile elements such as viruses or plasmids, or through the uptake of free or encapsulated DNA from the environment [12,13]. Genomes can acquire novel genes in a fashion ranging from transient to permanent depending on the type of element and the physiological conditions of the host. When ME succeed in stably inserting their genome, the inserted DNA is then replicated as part of the host chromosome. The transactions between ME DNA and host genome are catalyzed by recombinases typically encoded by the elements themselves. These recombinases rank in different classes based on their enzymatic activity and the specificity of their DNA targets. The smallest ME are insertion sequences (IS) composed of a short DNA segment encoding only the enzymes involved in their transposition which can occur at many different genomic locations [14]. The related transposons are larger DNA segments which can be transposed by two flanking IS and frequently carry additional genes such as antibiotic resistance determinants [15]. The most frequent IS recombinases are DDE transposases which do not form covalent transposase-DNA intermediates during transposition [16]. Other and typically larger ME such as plasmids and viruses encode recombinases promoting DNA transactions with a stronger DNA sequence specificity. Such site-specific recombination is not only used for mobile element integration and excision in bacteria but also in the spread of antibiotic resistance by transposable elements, the control of plasmid copy number, regulation of gene expression and the resolution of concatenated chromosomes [17]. Site-specific recombinases can be categorized into the serine recombinases and tyrosine recombinases (Y-recombinases); which, in contrast to DDE transposases, form covalent enzyme-DNA intermediates during recombination, albeit with markedly different mechanisms of action. Before religation of the two recombining DNA strands, serine recombinases generate breaks in all strands while Y-recombinases produce two sequential single-strand breaks [17]. As a rule, site-specific integration/excision reactions promoted by Y-recombinases occur via a synaptic complex composed of two DNA duplexes carrying the specific sites bound by four recombinase protomers [17]. The two-recombinase pairs are activated sequentially, allowing one strand from each duplex to be exchanged at a time via two consecutive and symmetrical Holliday junctions. A notable exception is *Vibrio cholerae* phage CTX. Not only does this phage integrate into its host genome in single stranded form where two sites fold into a hairpin structure, mimicking a recombination target for the cellular XerCD chromosome resolvase; but also only requires XerC for integration [18].

One of the best-studied Y-recombinases is the integrase of phage λ. The primary function of this enzyme is the integration of phage DNA into the chromosome of its bacterial host (and its excision). This function is achieved by promoting site-specific recombination between the phage attachment site *attP* and its chromosomal counterpart *attB* [19]. Under particular circumstances, the integrase of the lambdoid phage HK022 is capable of generating inversions between *attP* and a secondary attachment site in the HK022 left operon [3]. Similarly, the primary function of the yeast FLP protein is the control of the 2μ plasmid copy number [20] by DNA inversion between two divergent 34bp FRT sites located on the plasmid [21]. FLP recombinase activity has also been successfully used for integration and excision of synthetic DNA in mammalian genomes [22]. The recombination activities of both λ integrase and FLP recombinase are summarized as shown in S1 Fig. Historically, this reciprocal and conservative recombination between two stringently defined double-stranded DNA sequences in each chromosome was denominated the Campbell model [23].

The sequences of a considerable number of Y-recombinases have been compared to reveal the position of conserved residues and infer the location of the catalytic active site [24]. They share in their C-terminal moiety a rather well conserved region of ~120 amino acids containing up to six nearly invariant amino acids R..K..HxxR..[W/H]..Y forming the active site [25,26]. A small number of Y-recombinases have been characterized biochemically in Archaea, for example the XerA recombinase of the hyperthermophilic euryarchaeon *Pyrococcus abyssi* which exhibits a perfect active site consensus [27]. Sequence alignments have revealed that other archaeal active sites diverge slightly from the bacterial consensus R..HxxR..Y [28]. The integrases of viruses SSV1 isolated from the hyperthermophilic crenarchaeon *Sulfolobus shibatae* [29] and SSV2 from *Sulfolobus islandicus* [30] share the consensus R..KxxR..Y while the plasmidic integrase of *Sulfolobus* sp. NOB8H2 displays R..YxxR..Y [28].

Mobile elements therefore contribute to genome evolution through both site-specific and homologous recombination, which usually operate by distinct mechanisms and enzymatic activities. Homologous recombination is also known to occur frequently between multiple IS copies resulting in large scale archaeal genomic rearrangements, as observed in both *Crenarchaeota* e.g. *Sulfolobus islandicus* [31] and *Euryarchaota* e.g. *Pyrococcus abyssi* [32]. The distribution of archaeal ISs is patchy not only at the phylum level but also at genus level [9]. Interestingly, genome shuffling occurs in *Thermococcus* [33] even if ISs are seldom found in this genus suggesting that alternative recombination mechanisms are capable of producing large-scale genomic rearrangements.

If site-specific recombination only requires specific nucleotide sequences targeted by a dedicated recombinase, homologous recombination on the other hand is a much more complex process. In all organisms, homologous recombination constitutes one of several pathways to repair double-strand breaks. In addition to DNA synthesis, it requires dedicated recombinases and their accessory factors which act on stretches of near-sequence-identical DNA. In eukaryotic and bacterial cells, the enzymes and pathways involved in homologous recombination have been extensively studied (see [34,35] for reviews), whereas archaeal homologous recombination is still an active field of investigation. It is known that the initial resectioning step after double-strand break involves the Rad50–Mre11–HerA–NurA complex to generate 3’ single-strand substrates [36,37]. The RecA paralog RadA and its accessory functions associate with this ssDNA to constitute the presynaptic filament, which will scan and pair with homologous sequences [38]. In the archaeon *Thermococcus kodakarensis*, homologous recombination has been detected experimentally between stretches of identical DNA sequences equal to or greater than 500bp [39].

To our knowledge, a direct overlap between site-specific and homologous recombination processes has not been described so far. In the present work, we report the discovery and characterization of a new integrase from the hyperthermophilic archaeon *Thermococcus nautili* [40,41] capable of catalyzing both site-specific recombination and low sequence specificity recombination reactions mimicking homologous recombination. The wide distribution of this particular Y-recombinase among the *Thermococcus* genus provides a valid rationale for the observed genomic rearrangements in these Archaea.

## Results

### Dotplot comparisons identify synteny breakpoints in *Thermococcus* chromosomes

**Fig 1:**
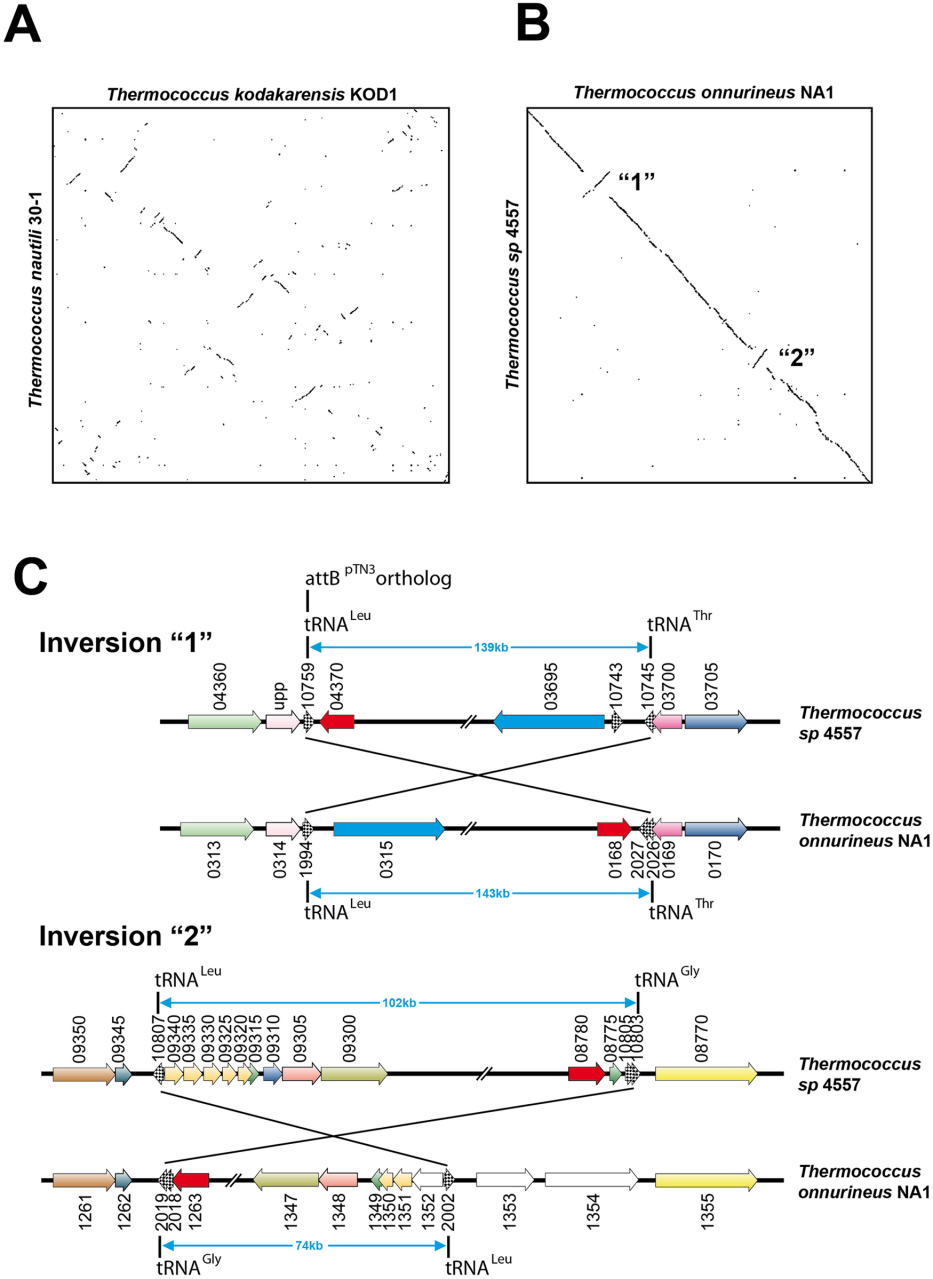
Genomic dotplots and synteny analysis. Genomic dotplots (**A**) between *T*. *kodakarensis* and *T*. *nautili* and (**B**) between *T*. *onnurineus* and *T*. *sp*. 4557. All genomes are centered on their putative predicted origin of replication [33]. **C.** The two synteny breaks in the genomic alignment between *T*. *onnurineus* and *T*. sp. 4557 (Panel B) were further analyzed. Gene order conservation and recombination endpoints of the two major inversions were identified using composite images generated by the SyntTax web tool. Inversion “1” occurred between tRNA^Leu^ (GQS_t10759) and tRNA^Thr^ (GQS_t10745) genes; *T*. sp. 4557 GQS_t10759 gene is orthologous to the *T*. *nautili* tRNA^Leu^ gene (BD01_0018) which corresponds to the chromosomal attachment site of plasmid pTN3. Inversion “2” (Panel B) occurred between tRNA^Leu^ (GQS_t10807) and tRNA^Gly^ (GQS_t10803) genes.

We compared the chromosomes of the 13 completely sequenced *Thermococcus* species available to date by dotplot analysis and observed high levels of genome scrambling as shown in Fig 1A. Strikingly, comparison of *T*. *onnurineus* and *T*. sp. 4557 chromosomes by this approach revealed only two large inversions of 139/143Kb and 102/74Kb respectively (Fig 1B & 1C). This relatively small number of inversions facilitated the investigation of the synteny breakpoints bordering both inversions. Using the SyntTax web tool [42], a composite representation was obtained as shown in Fig 1C. Gene order is conserved immediately upstream and downstream of each inversion border and was used to identify the synteny breakpoints. For each inversion, the breakpoints are located within tRNA gene pairs, transcribed in opposite orientations. Interestingly, *T*. *nautili* plasmid pTN3 integrates in the tRNA^Leu^ gene BD01_0018 [41,43] (S2 Fig) and this gene displays over 97% sequence identity with tRNA^Leu^ (GQS_t10759), which borders a large chromosomal inversion between *T*. *onnurineus* and *T*. sp. 4557 (Fig 1B). The concordance between the chromosomal attachment site of the pTN3 integrase (Int^pTN3^) and the recombination targets bordering each inversion (in opposite orientations) led us to define a working model to explain the formation of genomic inversions observed in the *Thermococcus* genus. We hypothesize that the frequent genomic inversions observed in the evolution of the *Thermococcales* order are a result of enzymatic activity of the integrase encoded by horizontally mobile elements, such as pTN3.

### Int^pTN3^ is a *bona fide* tyrosine recombinase

The integrase of pTN3 shares significant sequence similarity with canonical Y-recombinases and its predicted active site can be defined as R..K..AxxR..Y which only slightly diverges from the consensus (S3A Fig). In addition, Int^pTN3^ displays a high degree of conservation with two biochemically characterized hyperthermophilic Y-recombinases, the archaeal Int^SSV1^ [44] and Int^SSV2^ [30] (S3B Fig). Thus, it seemed worthwhile to compare the enzymatic activities of Int^pTN3^ to those of other enzymes of the same family such as phage λ integrase and *Saccharomyces cerevisiae* 2μ plasmid FLP protein and to validate them against the canonical Y-recombinase model.

### Int^pTN3^ is an active site-specific tyrosine recombinase

In order to characterize the activities of Int^pTN3^, it was necessary to over-produce and purify the enzyme (S4 Fig) and to construct DNA substrates carrying appropriate attachment sites (as determined by sequential deletions (S5 Fig). An integrase variant (Int^pTN3^Y428A) in which the catalytic tyrosine is substituted with an alanine was constructed, purified and tested (S6 Fig). We used these proteins and DNA components in a series of *in vitro* and *in vivo* experiments, detailed below, to ascertain the properties of Int^pTN3^.

#### Int^pTN3^ catalyzes *attP-attB* integration

**Fig 2:**
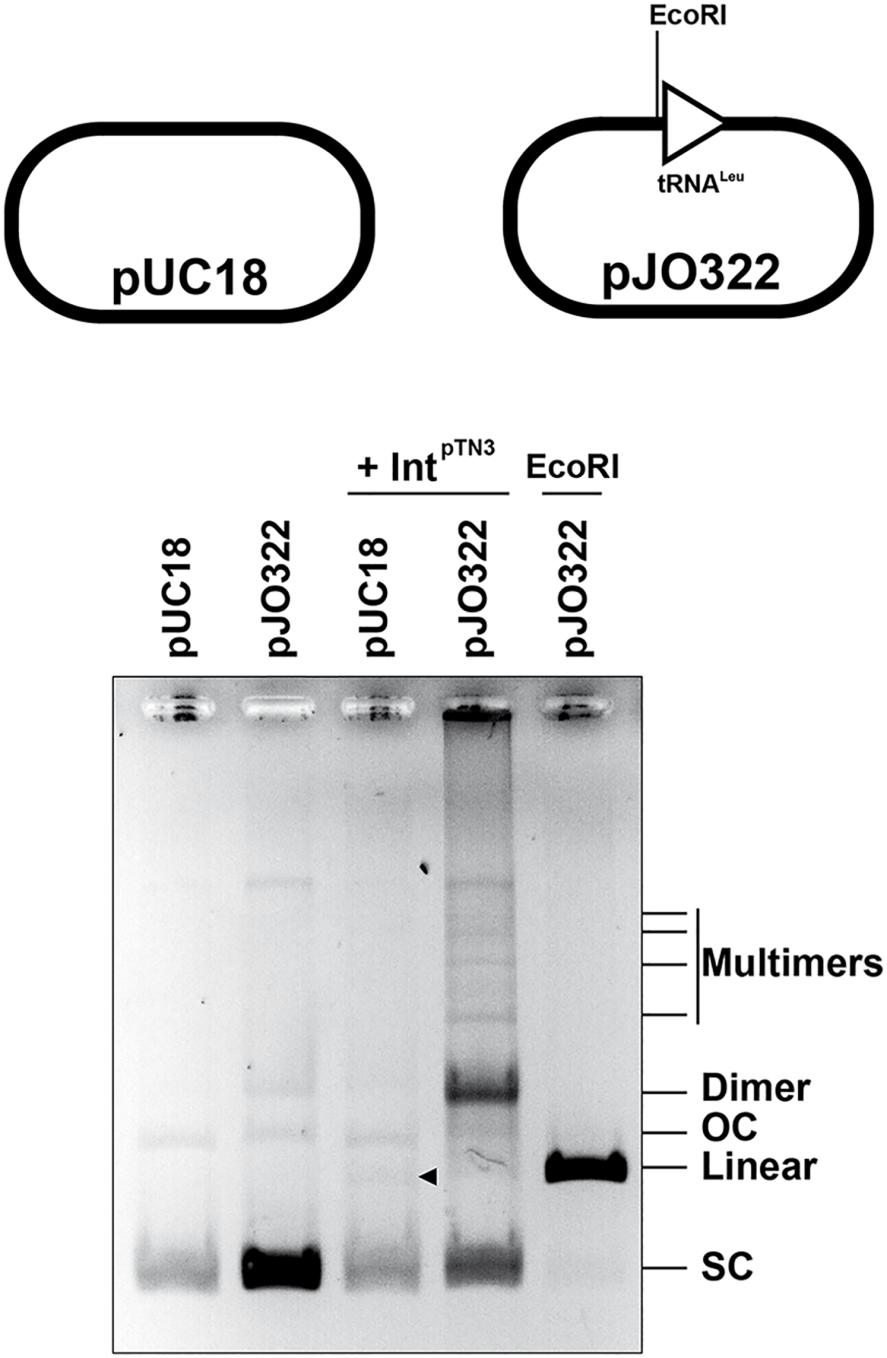
Dimer formation. Supercoiled (SC) plasmids pUC18 and pJO322 carrying the Leu2-88 fragment (S5A Fig) were incubated with Int^pTN3^ in a standard reaction (see Materials and methods) and compared with linearized pJO322 by agarose gel electrophoresis. The integrase has no effect on pUC18 with the exception of the production of a faint linear species (indicated by an arrow). The integrase increases considerably the formation of plasmid pJO322 dimers and to a lower extent that of multimers. No increase in the formation of open circular (OC) form was observed.

In order to measure the activity of purified Int^pTN3^, we initially developed a simple test in which integrase-catalyzed integration of one plasmid-encoded *attB* site in an identical site on a second plasmid results in formation of a plasmid-plasmid dimer (S1A Fig), which can be detected by gel electrophoresis. In accordance with our identification of tRNA^Leu^ as a potential *attB* site, we generated a supercoiled DNA template carrying a quasi-full-length *T*. *nautili* tRNA^Leu^ gene, Leu2-88 (see below). We observed the formation of dimeric DNA molecules only with DNA templates carrying *attB* tRNA^Leu^, and only in the presence of Int^pTN3^ (Fig 2). Thus, the Int^pTN3^ is able to catalyze the site-specific recombination of one *att* site with another.

#### Int^pTN3^ catalyzes *attL-attR* excision

**Fig 3:**
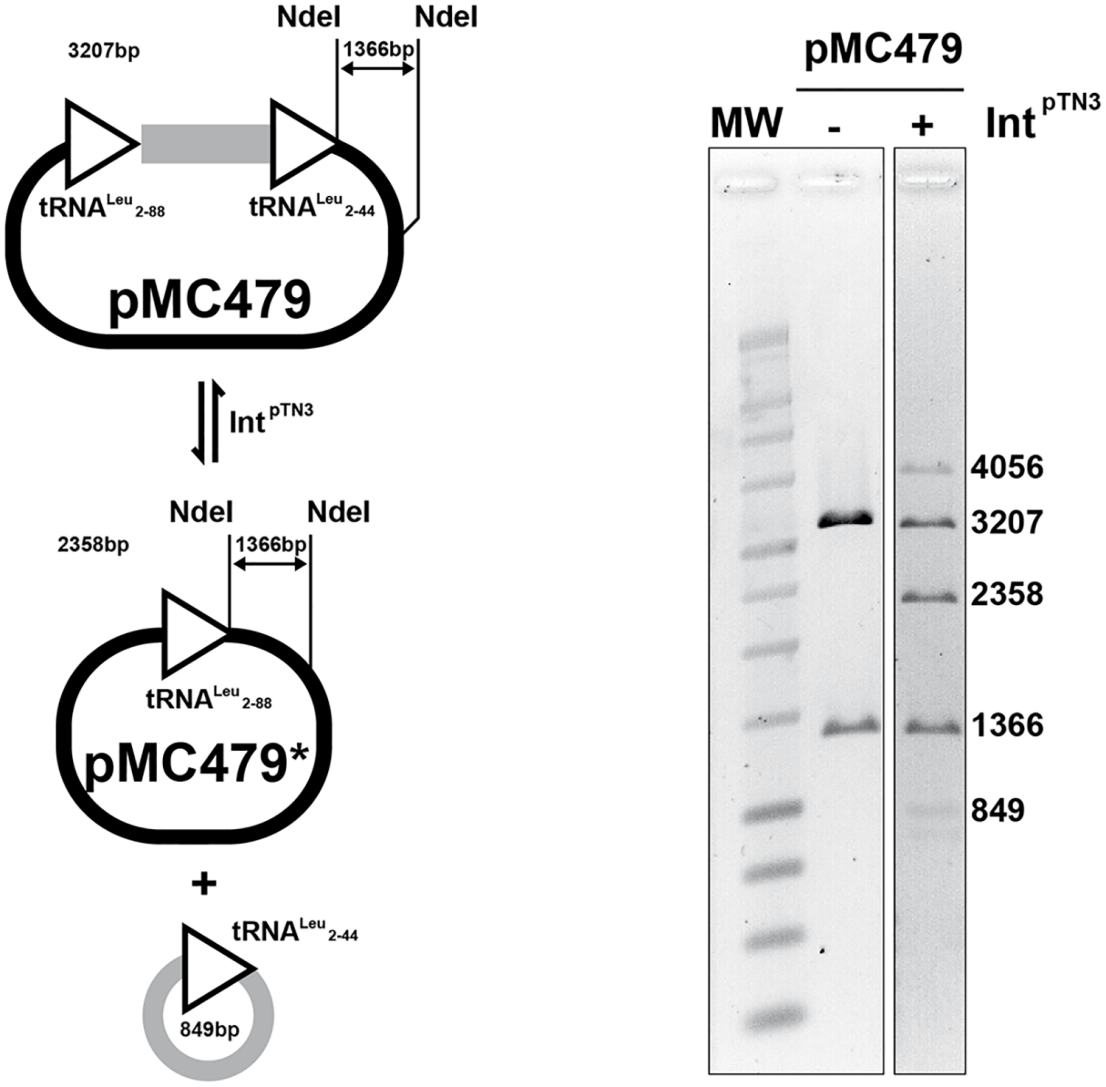
Int^ptn3^ excision and integration. Plasmid pMC479 carries two copies of tRNA^Leu^ cloned in direct orientation and separated by a 762bp spacer fragment (see Material and methods). The direct repeats consist of the minimal tRNA^Leu^ 2–44 and the longer tRNA^Leu^ 2–88, both proficient in dimerization reactions. Plasmid pMC479 was incubated with Int^pTN3^ in a standard reaction (see Materials and methods). The *Nde*I restriction enzyme generates two fragments of 3207 and 1366bp respectively in pMC479. Upon incubation with Int^pTN3^, *Nde*I digestion generates additional fragments of 2358bp corresponding to recombined pMC479* and 849bp corresponding to the circularized spacer and recombined *att* site. Both constitute the products of the excision reaction. A larger 4056bp fragment is generated as well and corresponds to the recombination product generated by integration of the 3207 and 849bp species. The relative intensity of the bands is compatible with an expected equilibrium reaction.

The capacity of Int^pTN3^ to catalyze the inverse reaction i.e. the excision of a DNA segment located between *attL* and *attR* sites was tested using the template pMC479, which carries a Leu2-88 site and a minimal Leu2-44 site in the same orientation, separated by a 762bp segment. In the presence of Int^pTN3^, the restriction digestion pattern revealed the presence of two bands of 2358 and 849bp, consistent with the excision of a circular DNA species between two *attB* sites (Fig 3). The recombination reaction also generated an additional band of 4056bp, explainable by the integration of the 849bp circular product into the initial pMC478 template. This demonstrates that Int^pTN3^ is able to efficiently catalyze both DNA integration and excision reactions.

#### Int^pTN3^ can re-activate related integrated mobile elements

**Fig 4:**
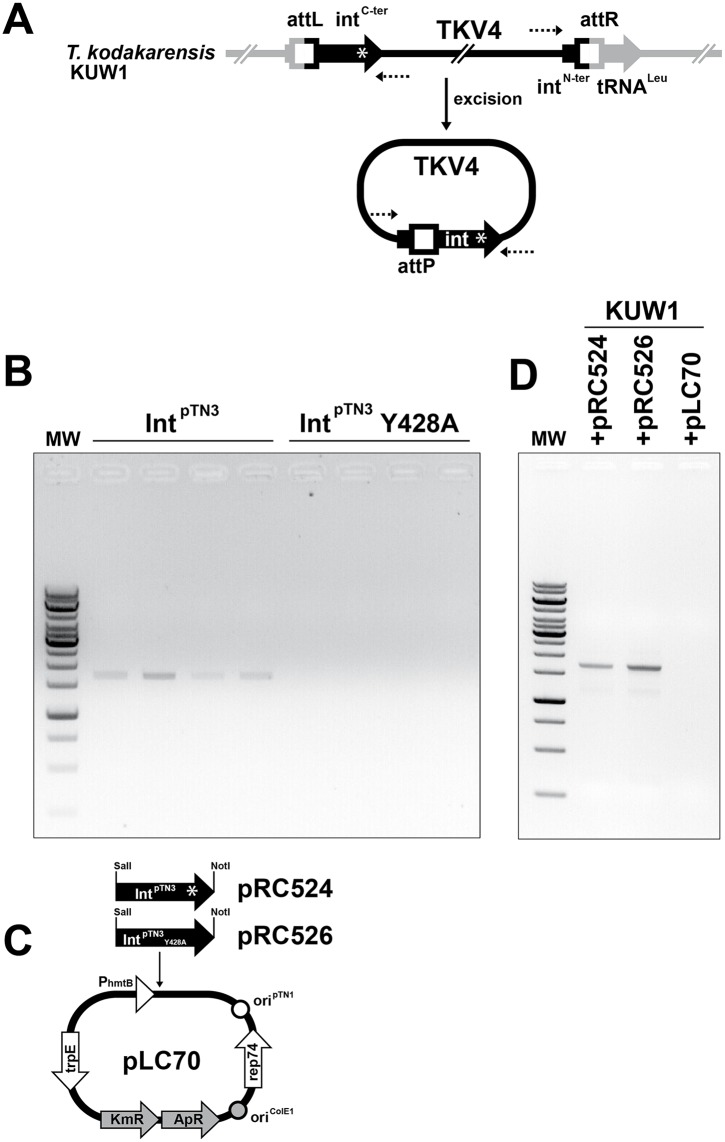
TKV4 excision *in vitro* and *in vivo*. A PCR amplification assay was designed to assert artificial Int^pTN3^-mediated TKV4 circularization (Panel A). The assay was first performed *in vitro* on four samples of purified *T*. *kodakarensis* genomic DNA incubated with wild type Int^pTN3^ or inactive Int^pTN3^ Y428A mutated enzyme in a standard reaction analyzed by agarose gel electrophoresis (see Materials and methods). Only reactions using wild-type enzyme generated a 1710bp band of the expected excision size (Panel B). The same TKV4 excision reaction was tested *in vivo* by transforming *T*. *kodakarensis* KUW1 with shuttle plasmids pRC524 (expressing wild type integrase) and pRC526 (expressing mutated Int^pTN3^Y428A) or with the vector alone (Panel C). Total DNA was extracted from the transformants and amplified as described above. In this *in vivo* experiment, both enzymes were TKV4 excision-proficient (Panel D).

The species *T*. *kodakarensis* carries in its genome the stably integrated element TKV4 [45], which is closely related to pTN3 of *T*. *nautili*. As shown for pTN3 (S2 Fig), the integration of TKV4 into the *T*. *kodakarensis* genome has disrupted the gene encoding Int^TKV4^, rendering TKV4 incapable of spontaneous chromosomal excision. Considering that Int^pTN3^ and Int^TKV4^ display extensive sequence similarity (S3 Fig) and promote integration in orthologous tRNA^Leu^ genes [45], we investigated the capacity of Int^pTN3^ to excise TKV4 *in vitro*. Excision and circularization of a DNA molecule is detectable by PCR amplification using suitably oriented primers (Fig 4A). Treatment of *T*. *kodakarensis* genomic DNA with purified Int^pTN3^ resulted in products consistent with TKV4 circularization (Fig 4B), demonstrating that Int^pTN3^ could excise, and hence re-activate this dormant mobile element. In light of this *in vitro* activity, we endeavored to test this TKV4 resurrection reaction *in vivo*. This experiment involved the construction of specialized *T*. *kodakarensis* expression vectors pRC524 and pRC526 expressing wild type Int^pTN3^ and mutant Int^pTN3^Y428A respectively (Fig 4C) (see Material and methods). Surprisingly, both Int^pTN3^ and the active site mutant Int^pTN3^Y428A were able to revive TKV4 *in vivo* (Fig 4D). Not only does this result demonstrate the ability of pTN3 to excise, and therefore re-activate integrated mobile elements, it also strongly suggests that the activity of mutated Int^pTN3^Y428A could be complemented by the truncated Int^TKV4^ encoded by the integrated element, whereas both variants are inactive on their own. A similar phenomenon of complementation has been reported between a DNA-binding impaired mutant and a catalytic tyrosine residue mutant of Int^SSV1^ [44].

#### Int^pTN3^ catalyzes DNA inversion between *att* sites

**Fig 5:**
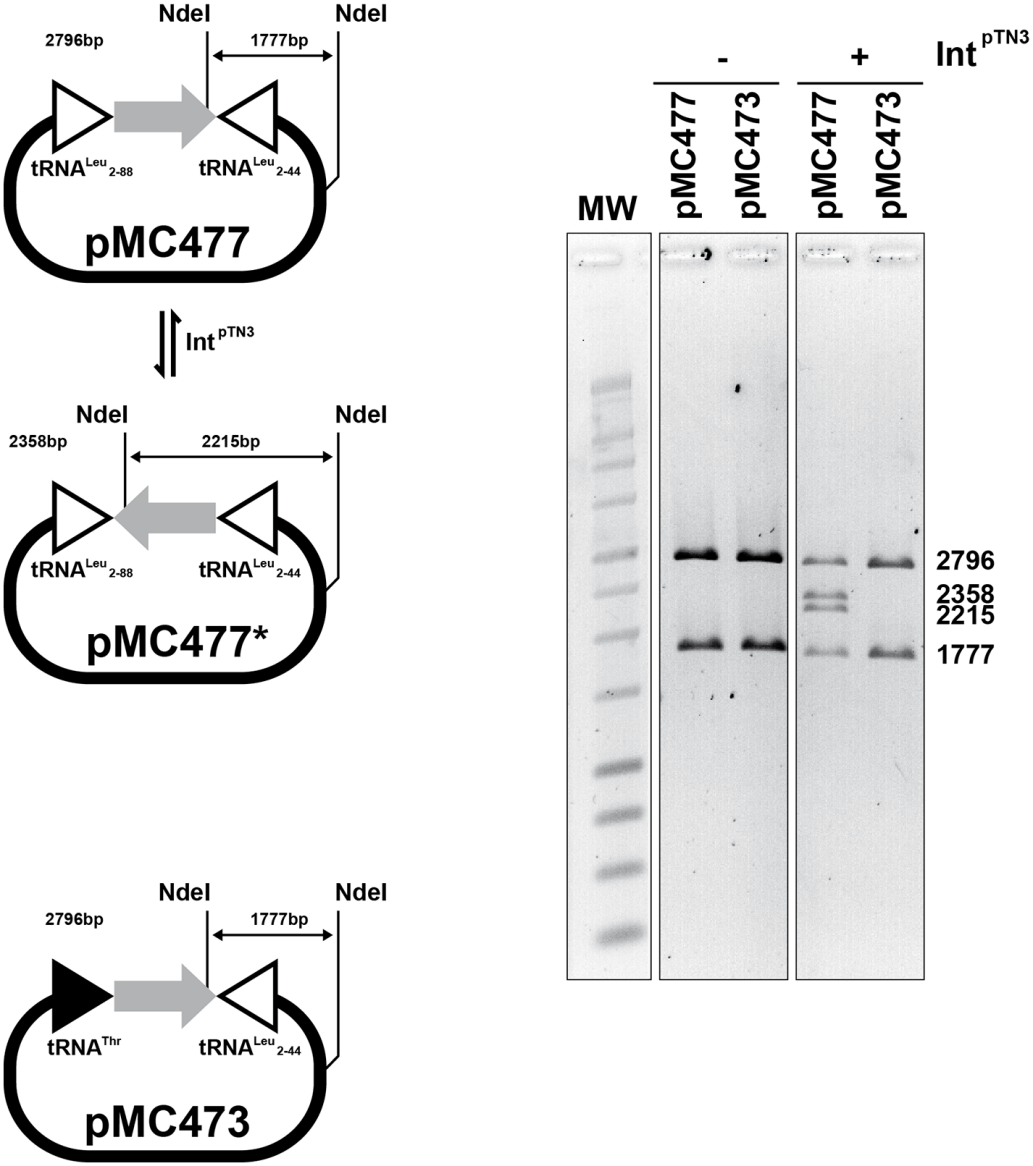
Int^ptn3^ inversion. Plasmid pMC477 carries two copies of tRNA^Leu^ cloned in inverted orientation and separated by a 892bp spacer fragment (see Material and methods). The inverted repeats consist of the minimal tRNA^Leu^ 2–44 and the longer tRNA^Leu^ 2–88, both proficient in dimerization reactions. Plasmid pMC473 carries tRNA^Leu^ 2–44 and tRNA^Thr^ GQS_t10745, in inverted orientation as well. Both plasmids were incubated with Int^pTN3^ in a standard reaction (see Materials and methods). The *Nde*I restriction enzyme generates in each case two fragments of 2796 and 1777bp. Upon incubation with Int^pTN3^, *Nde*I digestion of pMC477 generates additional fragments of 2358 and 2215bp corresponding to the recombinant pMC477*. As for the integration/excision reactions, the relative intensity of the bands is compatible with an expected equilibrium reaction. We could not detect any inversion between tRNA^Leu^ and tRNA^Thr^ in plasmid pMC473.

The ability of Int^pTN3^ to catalyze the inversion of DNA sequences is key in our model of large-scale integrase-mediated chromosomal rearrangements in the *Thermococcus* genus. To test the Int^pTN3^ invertase activity, we constructed a plasmid (pMC478) with two attachment sites in inverted orientation: the full-length tRNA^Leu^ gene and the minimal Leu2-44. The restriction digestion pattern showed the presence of two new bands corresponding to the inversion of the DNA segment between the *attB* sites only when DNA was treated with the integrase (Fig 5). This result indicates that, like the *S*. *cerevisiae* FLP recombinase, Int^pTN3^ is capable of efficiently performing all three canonical reactions characteristic of site-specific Y-recombinases: integration, excision and inversion. No recombination products could be observed in inversion reactions performed with the inactivated integrase variant Int^pTN3^Y428A (S6 Fig).

Synteny analysis of the inversion endpoints observed between *T*. sp. 4557 and *T*. *onnurineus* indicates that recombination may have occurred between different tRNA genes, namely between tRNA^Leu^ (GQS_t10759) and tRNA^Thr^ (GQS_t10745) as well as between tRNA^Leu^ (GQS_t10807) and tRNA^Gly^ (GQS_t10803). Interestingly, inversion templates combining tRNA^Leu^ and tRNA^Thr^ failed to produce recombination products (Fig 5).

### *Thermococcus nautili* undergoes rapid genomic rearrangement under laboratory conditions

**Fig 6:**
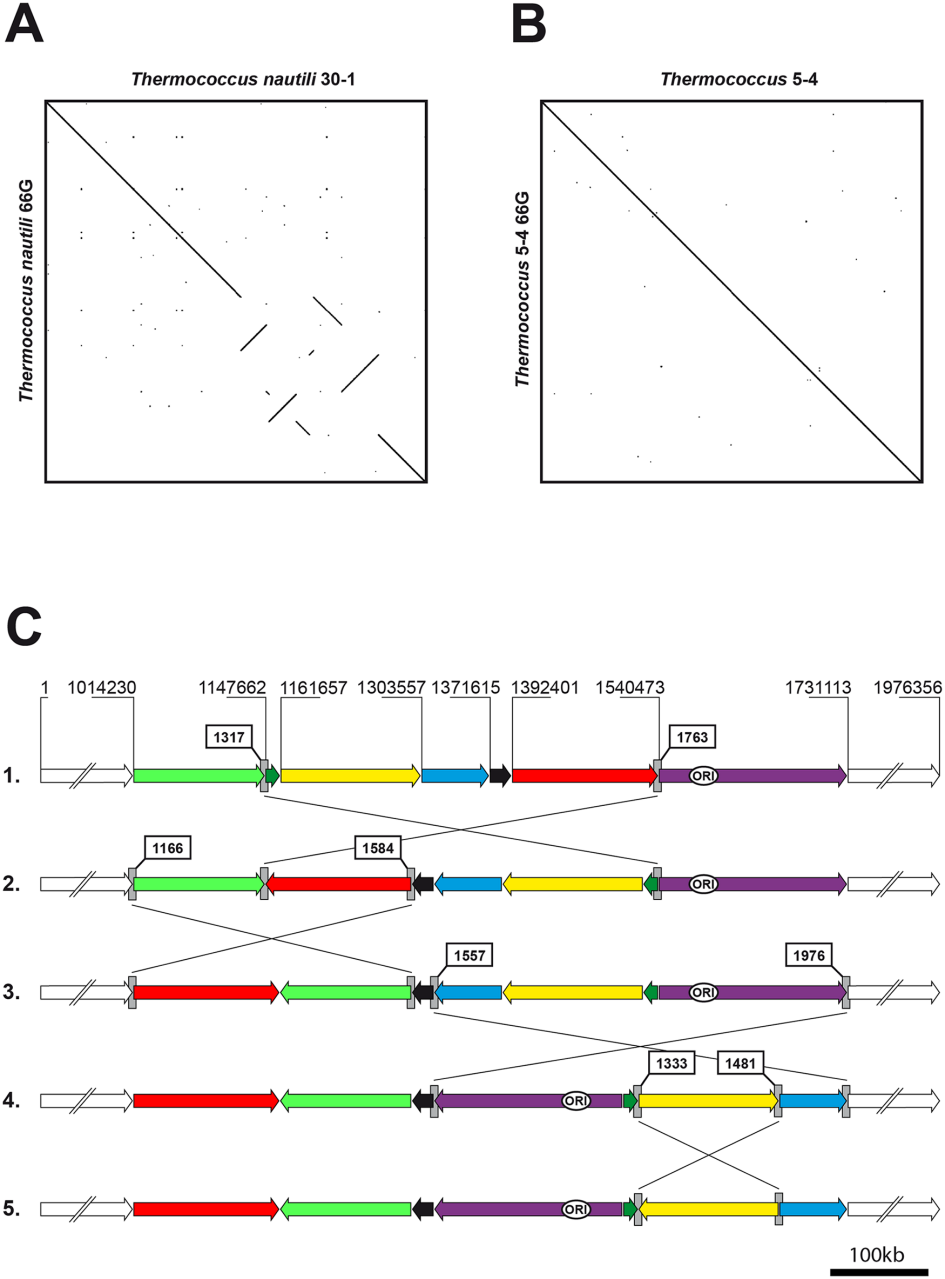
Laboratory inversions events. **A.** Dotplot analysis of the original isolate of *T*. *nautili* (GenBank accession NZ_CP007264) and the same organism after 66 generations (S2 Dataset). **B.** Dotplot analysis of the original isolate of *T*. 5–4 (GenBank accession CP021848) and the same organism after 66 generations (S4 Dataset). **C.** One of the possible sequential inversion scenarios leading to *T*. *nautili* 66G (Panel A), drawn to scale. The arrows direction reflects the chromosomal segment orientation in the original *T*. *nautili* strain. Genomic coordinates are indicated and the identifiers of the genes bordering each inversion are boxed.

The large-scale genomic inversions observed between *T*. sp. 4557 and *T*. *onnurineus* display minor gene order rearrangements near the recombination endpoints indicating that these events are not recent and might have undergone remodeling (Fig 1C). In order to identify more recent rearrangements, we investigated whether large-scale genomic inversions could occur spontaneously under laboratory conditions. *T*. *nautili* carrying its natural plasmids was sub-cultured in two independent experiments for 60 and 66 generations (therefore termed *T*. *nautili* 60G and 66G) in rich liquid medium with intermittent storage at 4°C and the metagenomes of the resulting populations were completely re-sequenced. We observed in both *T*. *nautili* 60G and 66G sub-cultures a high proportion of a novel rearranged genome exhibiting four new large-scale chromosomal inversions when compared to the original published *T*. *nautili* genome (GenBank accession NZ_CP007264) [41] (Fig 6A). By mapping the frequency of the Illumina reads around the four inversion sites, we measured the incidence of the rearranged genome in the *T*. *nautili* 66G population, which was found in most cases to exceed that of the original genome (S3 Table). Both *T*. *nautili* 60G and 66G rearranged chromosomes were remarkably similar when compared by dotplot analysis (S7 Fig). Additionally, plasmid pTN3 was largely underrepresented in the *T*. *nautili* 66G sub-culture (S3 Table), whereas the smaller pTN1 and pTN2 were conserved. The chromosomally-integrated pTN3 copy carrying the disrupted integrase gene was also retained. The chain of nested inversion events leading to these new recombined genomes could be reconstructed (Fig 6C) and allowed us to analyze and precisely map the recombination endpoints. Each of the four genomic inversions occurred between paralogous gene pairs: between tRNA^Gly^ genes BD01_1557 and BD01_1976, between methyl accepting chemotaxis genes BD01_1166 and BD01_1584, between transposase genes BD01_1317 and BD01_1763 and finally between UDP-glucose-6 dehydrogenase genes BD01_1333 and BD01_1481. For each pair of paralogous genes, the inversion events always occurred between two inverted segments of DNA sharing extensive sequence identity (S8 Fig). However, we could not detect significant similarity between inverted DNA segments corresponding to different pairs of paralogous genes using BLAST (e-value ≥ 0.075). Furthermore, none of these sequences could be aligned with the original pTN3 attachment site, tRNA^Leu^ (e-value ≥ 10). In a control experiment, in contrast to *T*. *nautili*, the genome of a closely related organism, the plasmid-less *Thermococcus sp*. 5–4 (GenBank accession CP021848) remained stable when sub-cultured for 36 or 66 generations in two separate experiments (Fig 6B and S7 Fig).

### Int^pTN3^ also catalyzes DNA inversion between non-*att* sites on the archaeal chromosome

**Fig 7:**
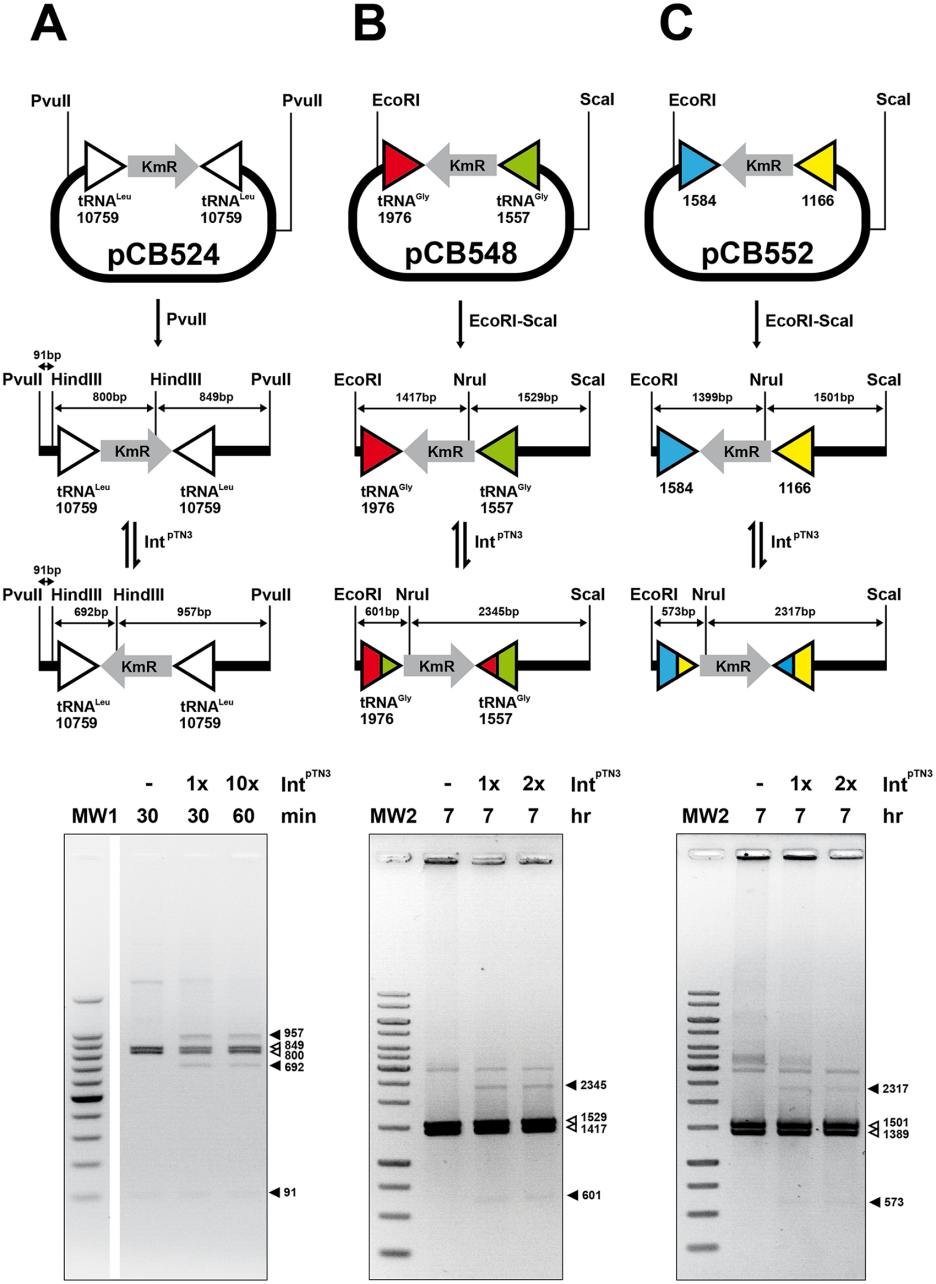
Int^pTN3^-promoted low sequence specificity reactions on archaeal sequences. Int^pTN3^ catalyzes inversion on linear DNA substrates between archaeal gene pairs separated by a Kanamycin resistance determinant. White arrowheads refer to original fragments and black arrowheads indicate inversions products. **A.** Inversion between two identical copies of tRNA^leu^ gene GQS_t10759 from *T*. *sp*. 4557. **B.** Inversion between tRNA^Gly^ genes BD01_1557 and BD01_1976 from *T*. *nautili*. **C.** Inversion between chemotaxis genes BD01_1166 and BD01_1584 from *T*. *nautili*. Int^pTN3^ concentration multipliers refer to the standard assay described in Materials and Methods. The detailed DNA sequences involved in these reactions are illustrated in S8 Fig.

The remarkable differences in the outcome of *T*. *nautili* and *T*. *sp* 5–4 sub-culturing experiments and the observation that tRNA^Gly^ genes could recombine in these conditions suggested a causal link between Int^pTN3^ and genome shuffling. To ascertain if the new recombinations in *T*. *nautili* 60G and 66G could have been indeed generated by Int^pTN3^, we decided to test whether this integrase was able to catalyze *in vitro* inversions using the sequences detected at the borders of these recombination events. New inversion templates pCB548 and pCB552 were thus constructed respectively carrying sequences encompassing tRNA^Gly^ genes BD01_1557 and BD01_1976 or sequence fragments from chemotaxis genes BD01_1166 and BD01_1584 (S8 Fig). To limit the number and size of generated fragments, an *in vitro* inversion assay was conducted on linear fragments originating from these plasmids and compared to a linear fragment carrying inverted *attP* sites derived from pCB524. Inversions could be detected with all three templates albeit with significantly longer incubation times or higher Int^pTN3^ concentrations for pCB548 and pCB552-derived templates as compared to pCB524 (Fig 7). To confirm this recombination event, one of the products of the pCB548 template inversion reaction was further characterized by DNA sequencing and corresponded to a *bona fide* cross-over between BD01_1557 and BD01_1976 (S9 Fig). We conclude that Int^pTN3^ is able to catalyze low sequence specificity recombination reactions between sites that differ in sequence from its cognate *att* site, with the same outcome as homologous recombination events. It is to be noted that Int^pTN3^ catalyzes these two types of reactions with a different efficiency. Site-specific recombination reactions reach the equilibrium within 30 minutes whereas several hours and higher enzyme concentrations are required to detect all low sequence specificity recombinations.

### Int^pTN3^ catalyzes low sequence specificity recombination reactions mimicking homologous recombination between any DNA sequence pairs

**Fig 8:**
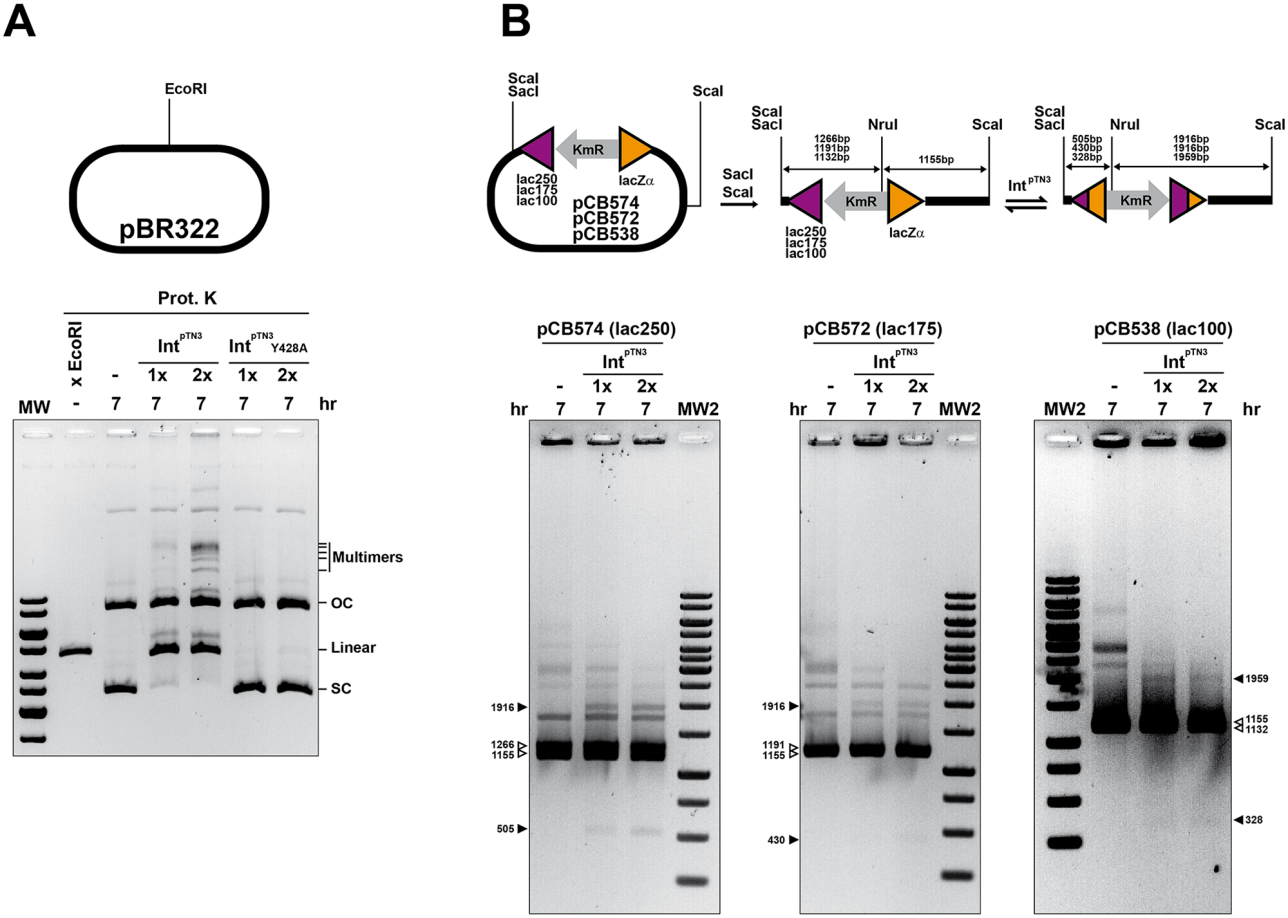
Int^pTN3^-promoted low sequence specificity reactions on exogenous sequences. **A.** Low sequence specificity reactions mimicking homologous DNA integration are visualized by the accumulation of multimers of increasing size only when the reaction occurs in the presence of wild-type Int^pTN3^. A linear pBR322 species generated by Int^pTN3^-generated double-strand cleavage is visible and migrates close to a control plasmid digested by the EcoRI endocnuclease. OC and SC refer to the open circle and supercoiled DNA forms, respectively. **B.** Int^pTN3^ catalyzes inversion on linear DNA substrates between two inverted *E*. *coli lacZ* gene segments of varying sizes separated by a Kanamycin resistance determinant. The sequence identity between the inverted segment amounts to 250, 175 and 100bp respectively in plasmids pCB574, pCB572 and pCB538 (see Materials and methods). White arrowheads refer to original fragments and black arrowheads indicate inversions products. Int^pTN3^ concentration multipliers refer to the standard assay described in Materials and Methods.

The absence of inter-pair DNA similarity observed in *T*. *nautili* 60G and 66G chromosomal inversions prompted us to test whether Int^pTN3^ could catalyze recombination between homologous non-archaeal sequences. The simplest experiment consisted of the incubation of cloning vector pBR322 DNA with the integrase in the same conditions as described above. This recombination reaction promoted by Int^pTN3^ yielded a ladder of plasmid multimers produced by sequential integration, which could be readily observed by eletrophoretic migration whereas no homologous integration reaction was detected with the mutated Int^pTN3^Y428A (Fig 8A). Surprisingly, Int^pTN3^ generated also a double-strand cut at the pBR322 ColE1 origin of replication for which we have no explanation at this stage (S10 Fig). This cleavage does not constitute an intermediate step in the recombination reaction since none of Int^pTN3^ linear substrates shown in Fig 7 carries the ColE1 origin. In addition to the homologous integration reaction, we investigated the capacity of Int^pTN3^ to promote inversions between homologous sequences of bacterial origin. Short DNA segments of decreasing length (250, 175 and 100bp, see S11 Fig) originating from the *E*. *coli lacZ* gene were cloned in opposite orientations respective to the *lacZα* gene of pUC18 to generate plasmids pCB574, pCB571 and pCB558, respectively. These templates were linearized, incubated with Int^pTN3^ and tested by subsequent restriction analysis. In each case, Int^pTN3^ generated additional bands consistent with homologous inversion reactions displaying efficiencies proportional to the extent of DNA identity (Fig 8B).

## Discussion

**Fig 9:**
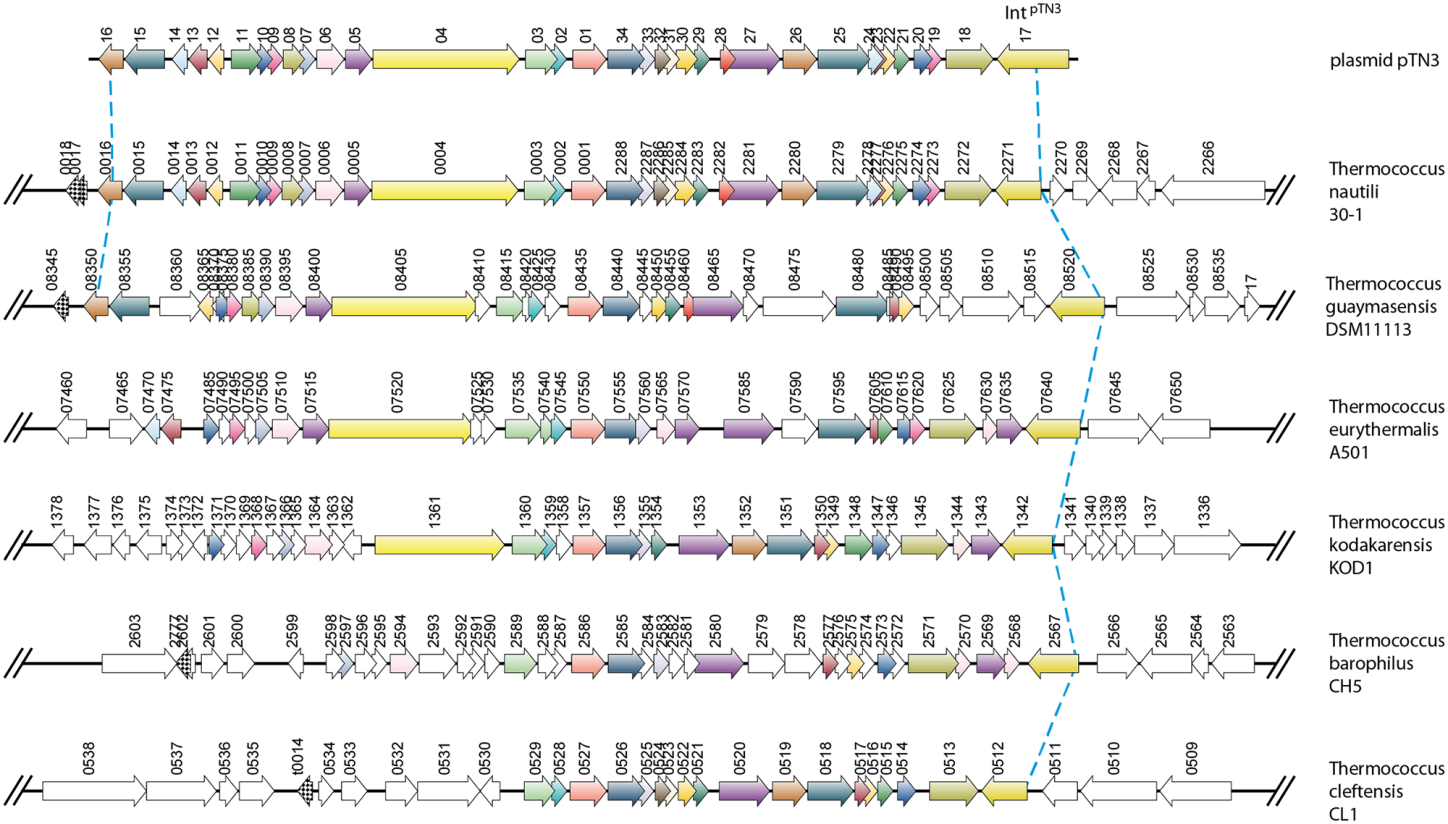
pTN3-like integrated elements in *Thermococcales*. The presence of pTN3-like integrated elements was investigated in all completely sequenced *Thermococcales* genomes by synteny analysis using the SyntTax web server [42]. In addition to *T*. *nautili*, the genomes of *T*. *guaymasensis* DSM11113, *T*. *eurythermalis* A501, *T*. *kodakarensis* KOD1, *T*. *barophilus* CH5, and *T*. *cleftensis* CL1 carry an extensive genomic region corresponding to plasmid pTN3 shown on top. Each arrow corresponds to an individual gene numbered according to GenBank annotations. The consistent gene color code illustrates orthology across organisms while white color indicates its absence. As indicated by a blue dotted line, conservation of synteny is clearly visible on the right border and limited by the gene encoding pTN3 C-ter integrase and its remnants. Truncated N-terminal-encoding integrase genes constitute pseudogenes lacking a stop codon and are therefore not annotated. Genetic divergence appears stronger on the left border.

The major mechanism producing chromosomal rearrangements is recombinational exchange between homologous sequences [46]. These rearrangements often consist of DNA inversions between IS elements [9,46,47]. The observation that, in the *Thermococcus* genus, large chromosomal inversions occur even in the absence of IS elements prompted us to investigate the molecular mechanism behind these rearrangements. The presence of tRNA genes at recombination endpoints in genomes as diverse as plant chloroplasts [48,49] and *Thermococcales* [9], combined with the fact that integrases often target tRNA genes [50], lead us to propose a precise molecular model involving Int^pTN3^ to explain large-scale genomic rearrangements. Using a combination of comparative genomics, *in vitro* analyses, and serial culturing experiments, we uncovered a mechanism and enzymatic activity responsible for the shuffling-driven chromosomal evolution in *Thermococcales*. By means of deep comparative genomic analyses, we were able to correlate genome scrambling with the presence of a mobile element. This mobile element has been identified as plasmid pTN3, naturally present in *T*. *nautili* both as an episome and integrated in the genome [41,43]. Plasmid pTN3 encodes the Int^pTN3^ integrase of the Y-recombinase superfamily capable of promoting its site-specific plasmid integration at a tRNA^Leu^ gene of its host. Due to perfect DNA conservation between *attB* and *attP* attachment sites (S2B Fig), an intact and presumably expressed tRNA^Leu^ is reconstituted upon pTN3 chromosomal integration. We successfully reproduced, with high efficiency in a purified *in vitro* system, the canonical DNA reactions of integration and excision expected from a *bona fide* integrase. Site-specific mutation of the active site tyrosine to alanine abolished these activities. A positive excision reaction was also obtained *in vivo* by expressing wild-type Int^pTN3^ and the catalytic tyrosine mutant Int^pTN3^Y428A in *T*. *kodakarensis* KOD1 cells. The genome of this strain carries the integrated episome TKV4 [45] which is remarkably similar to pTN3 (Fig 9). Surprisingly, both wild-type and mutant forms of the integrase excised TKV4 in circular form. This suggests that a truncated C-terminal Int^TKV4^, presumably impaired in DNA-binding but carrying the catalytic tyrosine, can complement Int^pTN3^Y428A. A plausible explanation invokes the participation of integrase dimers in the recombination reaction. In this case, only the heterodimeric form would possess an active catalytic site where Tyr428 is provided by the first monomer while the second monomer contributes the remaining conserved residues. This cleavage *in trans* was initially reported for the FLP recombinase [51,52]. Similarly, the complementation of activity between a DNA-binding impaired mutant and a catalytic tyrosine residue mutant has been described for another archaeal integrase, Int^SSV1^ [44].

The peculiar location of tRNA^Leu^ GQS_t10759 at the exact border of a large DNA inversion observed between the genomes of *T*. *onnurineus* and *T*. sp. 4557 suggested that this inversion could have occurred by the recombinase activity of Int^pTN3^. In our purified system, we could obtain highly efficient DNA inversions between two inverted copies of GQS_t10759. Paradoxically, we were unable to promote inversion between tRNA^Leu^ GQS_t10759 and tRNA^Thr^ GQS_t10745 contrary to what the genomic comparisons between *T*. *onnurineus* and *T*. sp. 4557 suggested. An experiment of prolonged *T*. *nautili* cultivation was instrumental in elucidating the large-scale inversion mechanism in *Thermococcus*. The strain carrying its natural plasmids was cultivated during 60 or 66 generations; total DNA was extracted from this population and sequenced in a manner similar to a metagenome. We observed the high incidence in the resulting populations of a particular recombined genome with four large chromosomal inversions and a very low copy number of plasmid pTN3 encoding active Int^pTN3^ (< 2/chromosome) (S3 Table). This plasmid loss could have contributed to the higher fitness and spread of a particular clone in the population. The four large-scale inversions occurred between four pairs of naturally occurring paralogous genes sharing at least 104bp of sequence identity in inverted orientation (S8 Fig). No significant sequence conservation could be detected between the four pairs. We did not observe chromosomal rearrangements after prolonged incubation of *Thermococcus* sp. 5–4, which does not carry plasmids. The potential causal link between pTN3 and a number of unrelated sequence pairs involved in large scale genomic shuffling in *T*. *nautili* was difficult to conciliate with the classical site-specific recombination properties we described for Int^pTN3^. Remarkably, by *in vitro* assays with this integrase, we succeeded in producing inversions between several pairs of inverted paralogous genes detected in our *T*. *nautili* sub-culturing experiments. These results suggested that the recombination properties of Int^pTN3^ could be extended to virtually any homologous pair of DNA sequences. Using exogenous pBR322 plasmid DNA or genes segments from bacterial origin, we demonstrated *in vitro* that Int^pTN3^ actively promotes low sequence specificity reactions mimicking homologous integration and inversion of any sequence pair as short as 100bp. The catalytic site mutation in variant Int^pTN3^Y428A abolishes this particular recombination reaction as well. Interestingly, cellular homologous recombination in Archaea operates according to a different pathway with dedicated enzymes [36,37] and in *Thermococcus kodakarensis* has only been reported between DNA segments of 500bp or more [39].

These reactions unveiled a specific Int^pTN3^-generated double-strand cut at the ColE1 origin of replication carried by pBR322 and its derivatives (S10 Fig). At this moment, we do not have a precise rationale to explain this observation other than a potential distant secondary structure similarity between the small RNAI and RNAII encoded by the ColE1 origin and the tRNA^Leu^ encoded by Int^pTN3^ *attB* substrate. Biological interactions between tRNAs and ColE1 RNAs have been reported [53]. Clearly, this double-strand cleavage does not participate in any recombination reaction since we demonstrated all *in vitro* Int^pTN3^ inversions on linear DNA segments devoid of ColE1 origin.

The positive *in vitro* Int^pTN3^-promoted low sequence specificity recombination results explain the failure of this enzyme to promote inversion between tRNA^Leu^ GQS_t10759 and tRNA^Thr^ GQS_t10745. These sites were initially thought to constitute inversion endpoints between the genomes of *T*. *onnurineus* and *T*. sp. 4557 but do not share sufficient sequence similarity to be efficiently recombined *in vitro*. The particular positioning of these sequences in opposite orientations could have occurred through previous overlapping inversions between a different set of paralogs or by less frequent native homologous recombination. We observed a similar situation in the sequence of the *T*. *nautili* 60G and 66G populations. In several cases, homologous segments were in direct orientation in the original genome but became opposed due to a previous overlapping inversion therefore indicating that *T*. *nautili* 60G and 66G inversions occurred sequentially.

In order to investigate whether pTN3 could account for large-scale rearrangements in the *Thermococcus* genus, we examined by synteny analysis the distribution of pTN3-like integrated element among completely sequenced *Thermococcales*. Out of 17 sequenced *Thermococcus*, and in addition to the previously reported *T*. *kodakarensis* TKV4 element [45], five isolates were found to harbor a pTN3-related element (Fig 9). The natural competence for DNA uptake of some *Thermococcales* such as *T*. *kodakarensis* [39] and the capacity of pTN3 to be transferred between cells using membrane vesicles [43] could explain the ubiquitous presence of this mobile element.

Protein sequence and structural comparisons between Int^pTN3^ and other hyperthermophilic archaeal integrases such as that of crenarchaeal virus SSV1 indicate that these proteins are clearly related. However, Int^pTN3^ possesses several additional interspersed domains relative to SSV1 (S2 and S12 Figs). We surmise that these additional domains contribute to the low sequence specificity recombination reactions akin to homologous recombination events that we have observed.

By summing up all direct and indirect evidence reported here, it is very likely that the integrase encoded by pTN3-like plasmids can account for the genomic shuffling observed in the *Thermococcus* genus. Plasmids of the pTN3 class are genetically closely related to viruses as they encode a capsid protein and a DNA packaging ATPase [43] but pTN3 virions have not be observed to date. It is not clear at this stage whether plasmids or viruses equipped with an Int^pTN3^-like integrase have a better fitness either due to provirus maintenance or by virion spreading. An integrase mimicking homologous recombination could promote viral integration into the host genome only if both viral and cellular chromosomes share significant DNA similarity. This enzyme however, could facilitate integration of a virus into the genome of a closely related provirus.

The question arises whether an enzyme promoting genome shuffling using very short repeated segments as substrates, would be beneficial for a cellular organism. On one hand, ‘wrongly’ recombined genomes would result in suboptimal gene expression programs and cells carrying scrambled genomes would display a reduced fitness and clearly be counter-selected in the population. Interestingly, the presence of a pTN3-specific spacer in a *T*. *nautili* CRISPR locus strongly suggests that the presence of this plasmid is deleterious [41]. On the other hand, it is also possible to envision situations where high-level genome shuffling by inversion could be advantageous. Alternate gene expression patterns could increase, for instance, adaptation to rapid environmental changes. In addition, for organisms such as *Thermococcales* where highly-expressed essential housekeeping genes maintain invariable positions [33], genome scrambling could be beneficial by relocating “less desirable” integrated elements to chromosomal areas of reduced gene expression, therefore minimizing their impact on cellular physiology.

## Materials and methods

### Bacterial, archaeal strains, plasmids and media

*Escherichia coli* strain XL1-Blue was used for cloning, plasmid amplification and site-directed mutagenesis. Overexpression of recombinant wild-type or mutant Int^pTN3^ was carried out in strain BL21 (DE3) (Novagen). All *E*. *coli* strains were grown in Luria-Bertani medium supplemented with 100μg/mL ampicillin or/and 50μg/mL kanamycin when necessary. *T*. *kodakarensis* KUW1 (*ΔpyrF ΔtrpE*) was grown anaerobically in ASW-YT medium [54] at 85°C. Long term *Thermococcus* sub-culturing experiments were carried out in the same conditions by sequential 50x dilutions of stationary phase cultures into fresh media. The number of generations was assessed statistically at each dilution step using a Thoma cell counting chamber under 400x magnification. The plasmids used or constructed in this work are listed in S1 Table. Transformation with pRC524 and pRC526 plasmids (see below) was performed following standard protocols [55]. Plasmid-containing KUW1 strains were grown in ASW-CH medium [54] supplemented with uracil (10 μg/mL). *T*. *nautili* sp. 30–1 (CP007264) was grown anaerobically at 85°C in Zillig’s broth [56].

### Bioinformatics and sequencing

Genomic sequences were compared and aligned by dotplot analysis using Gepard [57]. Conservation of gene order was assessed by synteny analysis using Absynte [58] and SyntTax [42]. The original genome of *Thermococcus* 5–4 JCM31817 (GenBank accession CP021848) and the genomes of sub-cultured *T*. *nautili* 60G and 66G and *T*. *sp*. 5–4 36G and 66G were sequenced by Genoscope (Centre National de Séquençage, France), using Illumina MiSeq. Reads were assembled with Newbler (release 2.9) and gap closure was performed by PCR, Sanger sequencing and Oxford Nanopore MinION. The primary genomic sequences of rearranged *T*. *nautili* 60G, 66G and *T*. 5–4 36G, 66G are available in S1, S2, S3 and S4 Datasets, respectively. These genomic sequences are compared by dotplot analysis in S7 Fig.

### Metagenome analysis

Genomic regions corresponding to ~2000bp upstream and downstream of inversion break-points were extracted from both the ancestral *T*. *nautili* sequence, and the sub-cultured *T*. *nautili* 66G sequence. Illumina sequencing reads were mapped to the ancestral sequence, and the pool of unmapped reads were mapped to the 66G sequence (Geneious 6.1.8). Two positions close to the break-point which differ in base composition between ancestral and 66G sequences were chosen to classify reads as resulting from original or inverted genome sequences. Bases were enumerated at these positions, and the percentage of reads corresponding to original sequences or inversions were calculated. The prevalence of pTN3 in the population was determined by comparing read depth across the entire *T*. *nautili* 66G genome (excluding the integrated pTN3 region) to that of pTN3 (S3 Table).

### Recombinant protein expression and purification

The gene encoding the integrase of the plasmid pTN3 of *T*. *nautili* 30–1, (gene ID: 17125032) was codon-optimized for expression in *E*. *coli* and synthesized by GenScript. The synthetic gene contained a Strep-Tag at the 5’ end and was cloned into pET26b+ expression vector (Novagen) to yield pJO344. Plasmid pJO496 carrying the mutated Int^pTN3^Y428A was obtained by site directed mutagenesis of pJO344 with primers Int_A and Int_B (S2 Table) using the Agilent QuikChange Lightning Site-Directed Mutagenesis Kit. Wild-type Int^pTN3^ and mutated Int^pTN3^Y428A were purified from *E*. *coli* BL21 (DE3) strain (Novagen) harboring respectively pJO344 or pJO496 by affinity chromatography and gel filtration (S4 Fig). All integrase enzymatic assays were conducted with strep-tagged protein derivatives.

### Integrase plasmid substrates

Plasmids used for the integrase dimerization assays were constructed as follows. *Eco*RI and *Bam*HI restriction sites were added respectively at the 5’ and 3’ end of the various oligonucleotides shown in S5 Fig. Each oligonucleotide (Sigma-Aldrich) was annealed to its complementary sequence and the resulting double-stranded segments were cloned between the corresponding restrictions sites of pUC18. To generate plasmid pMC451, the Leu2-88 fragment was cloned in pBR322 instead of pUC18. Plasmids pMC477 and pMC479 used respectively for *att* integration/excision and inversion assays were constructed using pMC451 as backbone. The insertion fragment was amplified with primers Leu43scaI_fw and Leu43scaI_rev using pMC449 plasmid DNA as template. It contains tRNA^Leu^ gene (2-44bp) and *lacZa* gene for blue-white screening. This amplified region was cloned in pMC451 in both possible orientation using *Sca*I and *Nru*I blunt sites. Plasmid pCB538 was obtained by amplifying with primers LacZ100-Sac1-For and KanR-Xba1-Rev (S2 Table) a 1364bp fragment from pUC4K and subsequent cloning between the XbaI-SacI sites of pUC18. The other plasmids: pCB548, pCB552, pCB572 and pCB574 used for non-att inversion assays were generated by Gibson Assembly [59]. Briefly, for pCB548, the genomic region corresponding to -80 to +245 of BD01_1557 (*T*. *nautili*) was amplified by PCR (Phusion Polymerase, ThermoScientific) using primers 1557_fwd and 1557_rev (S2 Table); the region from –80 to +245 of BD01_1976 was amplified using primers 1976_fwd and 1976_rev. The KmR gene was amplified from plasmid pUC4K using primers KanR_fwd and KanR_rev. Fragments were assembled into EcoRI + SalI digested pUC18 using the NEBuilder HiFi DNA Assembly Master Mix (New England Biolabs) following the manufacturer’s protocols. Similarly, for pCB552, part of the genes BD01_1166 and BD01_1584 (S8 Fig) were amplified by PCR and assembled into EcoRI + SalI digested pUC18 with the KmR gene sequence. To construct pCB538, a fragment containing KmR and the beginning of the *lacZ* gene (lac100) was PCR-amplified from pUC4K with the primers LacZ100-Sac1-For and KanR-Xba1-Rev containing the restriction sites for SacI and XbaI, respectively, at the 5’ end. The adequately digested fragment was then ligated into a SacI-XbaI digested pUC18. For plasmids pCB572 and pCB574, part of the *lacZ* gene was amplified from pUC18 and the KmR gene sequence was amplified from plasmid pUC4K. The two fragments were then assembled into the EcoRI digested pUC18. Purified plasmids pCB548, pCB552, were digested using ScaI and EcoRI and plasmids pCB572 and pCB574 were digested using ScaI. The fragments containing the non *att*-sites were then gel purified using the kit NucleoSpin Gel and PCR Clean-up (Macherey Nagel). All plasmid constructs were confirmed by DNA sequencing (Beckman Coulter Genomics).

### *In vitro/in vivo* integrase enzymatic assay

Standard *in vitro* integrase assays were performed as follows: 165ng (8.25ng/μL, 3.1pmol) purified Int^pTN3^ and 0.5μg (25ng/μL, 10pmol) supercoiled plasmid substrates were incubated 30 min at 65°C in a reaction buffer containing 300mM KCl, 27 mM Tris HCl pH8, 0.17mM DTT and 1mM MgSO_4_. Depending on the size of the plasmid substrate, the DNA/integrase molar ratio varied from 30 to 60. For substrates with non-*att* sites, the integrase concentration was increased up to 50pmol. To assay dimer formation, the reaction products were separated by gel electrophoresis and visualized with ethidium bromide. For the excision and inversion assays, reaction products were purified with the NucleoSpin Gel and PCR Clean-up kit (Macherey-Nagel) and digested with appropriate restriction enzymes (Thermo Scientific) prior to eletrophoretic separation. *In vitro* circularization of TKV4 was performed in a standard integrase assay with genomic DNA of *T*. *kodakarensis* isolated as described previously [60]. The reaction products were purified using NucleoSpin Gel and PCR Clean-up kit (Macherey-Nagel). Recircularized products were scored by amplifying a reconstituted full-length TKV4 integrase gene. PCR was performed using Phusion Polymerase (ThermoScientific) and primers TKV4_FW and TKV4_REV (S2 Table) in conditions recommended by the supplier. *In vivo* circularization of TKV4 was obtained using total DNA from *T*. *kodakarensis* KUW1 transformed with plasmid pRC524 or pRC526. These plasmids express constitutively wild type integrase and mutated Int^pTN3^Y428A from the P_hmtB_ promoter present in parental pLC70. DNA extraction and PCR reactions was performed as per the *in vitro* assay described above. To generate plasmids pRC524 and pRC526, the Int^pTN3^ integrase gene was amplified by PCR with primers int_fwd and int_rev (S2 Table), using total *T*. *nautili* genomic DNA as a template. The amplification product was cloned into pJET1.2 using the CloneJET PCR Cloning Kit (Thermo Fischer Scientific). The Y428A mutation was introduced into the integrase gene using the QuickChange II Site Directed Mutagenesis Kit (Agilent Technologies) with primer intY428A_fwd and its reverse complement. Both the wild-type and Y428A alleles were digested from pJET1.2 using *Sal*I and *Not*I and cloned into the corresponding sites of pLC70. All *in vitro* and *in vivo* recombination junctions and plasmid constructs were confirmed by DNA sequencing (Beckman Coulter Genomics).

## Supporting information

S1 Table Plasmids used in this work. (DOCX)

S2 Table Oligonucleotides used in this work. (DOCX)

S3 Table Metagenomic reads mapping *(T*. *nautili* 66G). (DOCX)

S1 Fig Classical site-specific recombination model. **A.** The intermolecular site-specific integration between cognate *attP* and *attB* sites generates a co-integrate with recombined *attL* and *attR* sites in direct orientation. The reverse reaction of excision regenerates the original components. **B.** In the intramolecular site-specific inversion reaction, the *att* sites are in opposite orientation. This reaction is reversible as well.
(PDF)

S2 Fig pTN3 integration. **A.** The comparison between the replicative and the chromosomal integrated forms of plasmid pTN3 enabled us to reconstitute the integration event. A stretch of 41bp is shared by both *attP* and *attB* sites. The nucleotides corresponding to the leucine anticodon are underlined. Upon integration, the integrase gene is disrupted and a full length tRNA^Leu^ gene is reconstituted although separated from its original promoter. An excision event would regenerate the original recombination partners. **B.** DNA sequence alignment between the integrase gene of pTN3 (black) and the tRNA^Leu^ gene (red). The start and stop codons of the integrase open reading frame are boxed in blue. The integration sites attP and attB as defined by Krupovic & Bamford [45] are boxed in their respective color.
(PDF)

S3 Fig Tyrosine recombinases sequence comparison. **A.** Alignment of Int^pTN3^ with tyrosine recombinases from the three domains of life. The protein sequence of Int^pTN3^ (WP_022547007.1) is aligned using Praline [Reference 4 in S1 Text] with the reconstituted integrase from *T*. *kodakarensis* TKV4 and other previously characterized tyrosine recombinases from the three domains of life. These recombinases consist of the integrases from Sulfolobus Spindle Viruses SSV1 (P20214.1) and SSV2 (NP_944456.1), phage λ integrase (ALA45781.1), phage HP1 integrase (NP_043466.1), XerD resolvase from *Escherichia coli* (NP_417370.1) and FLP recombinase from *Saccharomyces cerevisiae* 2μ plasmid (P03870.1). The region corresponding to the catalytic signatures (BoxI, K_β_, BoxII) of crystallized tyrosine recombinases are boxed in light gray. The predicted residues composing Int^pTN3^ catalytic site are shown (R..K..AxxR..Y) and the catalytic tyrosine residue is indicated by a black arrow. The color code refers to the extent of residue conservation at each position as show in the color scale. **B.** Alignment of Int^pTN3^ with Int^TKV4^ and the hyperthermophilic tyrosine recombinases Int^SSV1^ and Int^SSV2^. Global protein sequence similarities were computed with the Needleman-Wunsch algorithm (Needle EMBOSS, http://www.ebi.ac.uk/Tools/psa/emboss_needle/): Int^pTN3^-Int^TKV4^: 93.6%; Int^pTN3^-Int^SSV1^: 33.0% and Int^pTN3^-Int^SSV2^: 31.2%.
(PDF)

S4 Fig Int^ptn3^ overexpression and purification. **A.** Protein expression was induced with 1mM IPTG in 1L of LB medium; cells harvested by centrifugation, and lysed by sonication. The soluble fraction of the sonicate was heated at 65°C for 10 minutes, and denatured proteins removed by centrifugation and by passing through a 0.45 μm filter. Strep-tagged proteins were purified by affinity fractionation using a Strep-Tactin column (IBA Lifesciences) as recommended by the supplier. **B.** Strep-Tactin fractions 4 and 5 were pooled and submitted to gel filtration (Superdex 200 16/600, GE Healthcare). **C.** Gel filtration fractions 21 to 31 were pooled and the purified protein was concentrated with an Amicon 3kDa cutoff concentrator (Millipore), aliquoted and stored at -80°C.
(PDF)

S5 Fig AttB nested deletions. The Integrase dimerization test was used to determine the minimal site required for Int^pTN3^ tRNA^Leu^ × tRNA^Leu^ recombination on nested deletions carried by plasmid templates. **A.** DNA sequence of the nested deletions. DNA segments corresponding to theses sequences were annealed and cloned directionally in pUC18. **B.** The resulting supercoiled plasmids were incubated with purified Int^pTN3^ in a standard reaction and scored for dimer formation by agarose gel electrophoresis where only relevant reactions are shown. The dimerization-proficient sequences in Panel A are marked as positive. It is noteworthy that the Leu41 site, a site corresponding to the 41bp of sequence identity shared by both *attP* and *attB* is not a sufficient substrate for this reaction. Therefore, the minimal site for efficient dimerization is Leu2-44 with a size of 43bp. The asterisks indicate the extent of sequence identity between chromosomal *att*B and pTN3 *att*P. The leucine CAA anticodon is underlined.
(PDF)

S6 Fig Mutated IntY428A assay. Increasing amounts of wild type Int^pTN3^ and mutated Int^pTN3^Y428A enzymes were incubated with plasmid pMC477 as substrate to analyze the inversion properties. The experimental conditions are those of the standard integrase assay (see Material and methods) except that increasing amounts of enzyme were used: 0.5, 1, 1.5, 2.5 and 5μg, respectively. No inversion is detectable with Int^pTN3^Y428A.
(PDF)

S7 Fig Subcultures genome comparisons. Dotplot alignment of the prominent genomes obtained after *T*. *nautili* 60G and 66G subculturing (left) and *T*. 5–4 36G and 66G (right).
(PDF)

S8 Fig Detailed mapping of the Int^pTN3^-promoted *in vivo* inversions between four pairs of *T*. *nautili* paralogs. The sequences corresponding to the four genomic crossovers observed in *T*. *nautili* 60G and 66G were identified each time in pairs of paralogous genes shown aligned here. The sequences blocked in grey throughout the figure refer to perfectly conserved DNA segments in each paralogous pair where recombination occurred. Short sequences boxed in red refer to open reading frames start and stop codons when applicable (see also Fig 7 for throughout consistent color-coding). **Panel A** shows the alignment between segments overlapping tRNA^Gly^ genes BD01_1557 and BD01_1976. The precise regions corresponding to both tRNA^Gly^ genes are boxed in black. DNA segments cloned in pCB548 indicated by green blocks refer to BD01_1557-related sequences while red blocks correspond to BD01_1976-related sequences. The BD01-1976 nucleotide highlighted in black corrects a sequencing error in the original *T*. *nautili* genome sequence. A 176bp segment (grayed) is perfectly conserved between BD01_1557 and BD01_1976. Gly anticodons are boxed in yellow color. **Panel B** displays the alignment between methyl accepting chemotaxis genes BD01_1166 and BD01_1584. DNA segments cloned in pCB552 indicated by yellow blocks refer to BD01_1166-related sequences while blue blocks correspond to BD01_1594-related sequences A 176bp segment (grayed) is perfectly conserved between BD01_1166 and BD01_1584. **Panel C** displays the alignment between UDP-glucose-6 dehydrogenase genes BD01_1333 and BD01_1481. The two separate regions of extended sequence identity (I and II) are found between these genes respectively 284 and 620bp long (greyed). The presence of gene conversion in the interval between these two regions suggests that both were presumably involved in distinct crossover events. **Panel D** shows the alignment between transposase genes BD01_1317 and BD01_1763. The shortest recombination segment (104bp, grayed) is shared between these two paralogous genes.
(PDF)

S9 Fig Detailed characterization of Int^pTN3^-promoted *in vitro* inversion event by DNA sequencing. Specific sequences surrounding tRNA gene BD01_1976 are blocked in red while specific sequences surrounding tRNA gene BD01_1557 are blocked in green. Relevant anticodon sequences are boxed in yellow color. Two nucleotide mismatches between these tRNA genes are blocked in black. The tripartite composition of these DNA segments is further highlighted by blocking in grey color the stretch of identical sequenced shared by the DNA fragments carrying BD01_1976 and BD01_1557. **Panel A** depicts the sequence of steps involved in generating a suitable recombinant fragment for DNA sequencing. Plasmid pCB548 carries DNA segments containing *T*. *nautili* tRNA^Gly^-encoding genes BD01_1976 and BD01_1557 in inverted orientation and separated by a Kanamycin resistance determinant originating from pUC4K. The exact sequence of the cloned DNA segments encompassing BD01_1976 & BD01_1557 is displayed in S8A Fig. The inversion reaction was performed as shown in Fig 7B: an EcoRI-ScaI fragment originating from pCB548 was incubated with Int^pTN3^ after which the 601bp EcoRI-NruI fragment generated by Int^pTN3^ recombination was gel-purified, PCR-amplified with the forward primer 5’-ccgtttaatcgtcgcgcggaagc-3’ targeting the upstream sequence of the tRNA^Gly^ gene BD01_1976 and the reverse primer 5’-cccgttgaatatggctcataacaccc-3’ targeting the beginning of the KanR cassette. The resulting fragment was submitted to Sanger DNA sequencing using the forward primer mentioned above. **Panels B** and **C** display also the alignment between the 5’ half of both tRNA genes and the minimal Leu2-44 segment involved in Int^pTN3^ site-specific recombination. **Panel D** shows the result of the DNA sequencing reaction. The crossover point in the recombination reaction occurred precisely downstream of the two nucleotide mismatches mentioned above, in the sequence blocked in grey corresponding to the 3’ half of the tRNA genes and strictly conserved sequences immediately following. The sequences boxed in black in Panels B,C and D correspond to the exact extents of tRNAs^Gly^.
(PDF)

S10 Fig Integrase-promoted double-strand cut at ori ColE1. Circular plasmid pCB548 (4675bp) treated with Int^pTN3^ and digested with XhoI-NdeI endonucleases generates bands of 2966 and 1709bp due to integrase-promoted low sequence specificity recombination (white arrowheads). The original larger 3896bp XhoI-NdeI fragment undergoes an additional double-stranded cut at the plasmid ColE1 origin of replication to generate fragments of ~2400 and ~1500bp (black arrowheads). Int^pTN3^ concentration multipliers refer to the standard assay described in Materials and Methods.
(PDF)

S11 Fig *LacZ* gene segments used for low sequence specificity reactions mimicking homologous recombination. DNA sequence of the *lacZ* gene segments cloned in plasmids pCB538 (lac100), pCB572 (lac175) and pCB574 (lac250) (Fig 8B).
(PDF)

S12 Fig Integrase structure comparisons. The catalytic domain of Int^pTN3^ (**B**) was modeled using Phyre2 [Reference 5 in S1 Text] and compared using PyMol (The PyMOL Molecular Graphics System, Version 1.8 Schrödinger, LLC.) with the tridimensional structure of the integrase of *Sulfolobus solfataricus* virus SSV1 (PDB 3VCF) (**A**) determined by Zhan et al [Reference 6 in S1 Text]. The Int^pTN3^ catalytic tyrosine residue is highlighted.
(PDF)

S1 Dataset *Thermococcus nautili* 60G nucleotide sequence. Predominant *T*. *nautili* chromosome sequence obtained after sub-culturing for 60 generations.
(FASTA)

S2 Dataset *Thermococcus nautili* 66G nucleotide sequence. Predominant *T*. *nautili* chromosome sequence obtained after sub-culturing for 66 generations.
(FASTA)

S3 Dataset *Thermococcus* 5–4 36G nucleotide sequence. Predominant *T*. 5–4 chromosome sequence obtained after sub-culturing for 36 generations.
(FASTA)

S4 Dataset *Thermococcus* 5–4 66G nucleotide sequence. Predominant *T*. 5–4 chromosome sequence obtained after sub-culturing for 66 generations.
(FASTA)

S1 Text Supporting information references. (DOCX)
