# Supplementary material for: Flipping chromosomes in deep-sea archaea
Source: PLoS Genet. 2017 Jun 19;13(6):e1006847. doi: 10.1371/journal.pgen.1006847 (PMC5495485; doi:10.1371/journal.pgen.1006847)
Supplement: S1 Table — (DOCX) [file pgen.1006847.s001.docx]

**S1 Table. Plasmids used in this work**

| **Name** | **Backbone** | **Insertion** | **Selection** | **Reference** |
| --- | --- | --- | --- | --- |
| **pUC18** | - | - | ApR | [[1](#_ENREF_1)] in S1 Text |
| **pBR322** | - | - | ApR, TcR | [[2](#_ENREF_2)] in S1 Text |
| **pUC4K** | - | - | ApR, KmR | [[3](#_ENREF_3)] in S1 Text |
| **pET-26b+** | pBR322 | - | KmR | Novagen®, USA |
| **pLC70** | pCR2.1-TOPO  pTN1 | - | ApR, KmR, trpE, HMG-CoA red. | [[54](#_ENREF_4)] |
| **pJO344** | pET-26b+ | integrase gene from plasmid pTN3 | KmR | This work |
| **pJO496** | pJO344 | Int^pTN3^ Y428A allele | KmR | This work |
| **pJO322** | pUC18 | tRNA^Leu^ gene (2-88bp) from *T. nautili* | ApR | This work |
| **pMC451** | pBR322 | tRNA^Leu^ gene (2-88bp) from *T. nautili* | ApR, TcR | This work |
| **pMC449** | pUC18 | tRNA^Leu^ gene (2-44bp) | ApR | This work |
| **pMC477** | pMC451 | tRNA^Leu^ gene (2-44bp)+lacZα from pMC449 | ApR | This work |
| **pMC479** | pMC451 | tRNA^Leu^ gene (2-44bp)+lacZα from pMC449 | ApR | This work |
| **pRC524** | pLC70 | integrase gene from plasmid pTN3 | ApR, KmR, trpE, HMG-CoA red. | This work |
| **pRC526** | pLC70 | Y428A mutant of integrase gene from plasmid pTN3 | ApR, KmR, trpE, HMG-CoA red. | This work |
| **pCB538** | pUC18 | LacZ100 inverted fragment | ApR, KmR | This work |
| **pCB548** | pUC18 | genomic region of tRNA^Gly^ (gene ID: BD01_1557) + genomic region of tRNA^Gly^ (gene ID: BD01_1976) | ApR, KmR | This work |
| **pCB552** | pUC18 | genomic fragment of BD01_1166 + genomic fragment of BD01_1584 | ApR, KmR | This work |
| **pCB572** | pUC18 | LacZ175 inverted fragment | ApR, KmR | This work |
| **pCB574** | pUC18 | LacZ250 inverted fragment | ApR, KmR | This work |
