# Supplementary material for: Flipping chromosomes in deep-sea archaea
Source: PLoS Genet. 2017 Jun 19;13(6):e1006847. doi: 10.1371/journal.pgen.1006847 (PMC5495485; doi:10.1371/journal.pgen.1006847)
Supplement: S2 Table — (DOCX) [file pgen.1006847.s002.docx]

**S2 Table. Oligonucleotides used in this work.**

| Name | Sequence 5’-3’ | Usage | Description |
| --- | --- | --- | --- |
| LacZ100-Sac1-For | ctgacgtcctattacgccagctggcgaaagg | pCB538 | PCR amplification lacZ100-KmR |
| KanR-XBA1-Rev | gcgtctagaagccacgttgtgtctcaaaatctctg | pCB538 | PCR amplification lacZ100-KmR |
| 1557_fwd | agtgccaagcttgcatgcctgcaggtcgacGGTAGCTCAGCCTGGGAGAG | pCB548 | Gibson cloning |
| 1557_rev | acacaacgtggctAGTAAGTGAGGAGTGAAGCTCCAC | pCB548 | Gibson cloning |
| KanR_fwd | ctcctcacttactAGCCACGTTGTGTCTCAAAATC | pCB548 | Gibson cloning |
| KanR_rev | atctgctctacttCGCTGAGGTCTGCCTCGT | pCB548 | Gibson cloning |
| 1976_fwd | gcagacctcagcgAAGTAGAGCAGATTTTGCTCataaatcg | pCB548 | Gibson cloning |
| 1976_rev | ggaaacagctatgaccatgattacgaattcAATCGTCCGTTTAATCGTCgc | pCB548 | Gibson cloning |
| 1166_fwd-GIBSON | agtgccaagcttgcatgcctgcaggtcgacGAGCACCGAGAAGGGCGT | pCB552 | Gibson cloning |
| 1166_rev-GIBSON | acacaacgtggctGGTCAGAAGAAAAGGAAAATACGAG | pCB552 | Gibson cloning |
| KanR_fwd-GIBSON | ttttcttctgaccAGCCACGTTGTGTCTCAAAATC | pCB552 | Gibson cloning |
| KanR_rev-GIBSON | tttgagggggtgacgctgaggtctgcctcgt | pCB552 | Gibson cloning |
| 1584_fwd-GIBSON | gcagacctcagcgTCACCCCCTCAAAGTGGAAAGG | pCB552 | Gibson cloning |
| 1584_rev-GIBSON | ggaaacagctatgaccatgattacgaattcGAGCACCCAGCGCGGTGT | pCB552 | Gibson cloning |
| GRep-KanR-F | atccccgggtaccgagctcgAAGCCACGTTGTGTCTCAAAATC | pCB572/574 | Gibson cloning |
| GRep-KanR-R | acggccagtgCGCTGAGGTCTGCCTCGT | pCB572/574 | Gibson cloning |
| GRep-Repeat-F | gacctcagcgCACTGGCCGTCGTTTTAC | pCB572/574 | Gibson cloning |
| GRep-Lac175-ScaI_F | cagctatgaccatgattacgAGTACTAAATACCGCATCAGG | pCB572 | Gibson cloning |
| GRep-Lac250-ScaI_R | cagctatgaccatgattacgAGTACTTATGCGGCATCAG | pCB574 | Gibson cloning |
| Int_A | cacgttccaacgagcattggcgaccgccaacgtttttcggg | Mutagenesis  pJO496 | The Y428A mutation is underlined |
| Int_B | cccgaaaaacgttggcggtcgcaatgctcgttggaacgtg | Mutagenesis  pJO496 | The Y428A mutation is underlined |
| int_fwd | GATCGTCGACagcgatatatttatatagggatatagtaatagataatatcacaggtggtataga**ATGGTAAAATCGGGTGGTGTGTACG** | pRC526/548 | Bold nucleotides refer to the coding sequence of the integrase gene, underline indicates SalI and NotI sites, and lower case indicates PhmtB promoter sequence added for expression in *T. kodakarensis* |
| int_rev | GATCGCGGCCGC**TAAAGCTCCAGAATCCCCAGC** | pRC526/548 | idem |
| intY428A_fwd | CGTGGGTGGCAGGAACGCCCGCTGGAACGTCAAAAACG | pRC526/548  mutagenesis | The Y428A mutation is underlined |
| TKV4_FW | catgtgtcgttcctggtcgg | *In vitro/in vivo* excision assay |  |
| TKV4_REV | gggaggtaagacgggtaaggc | *In vitro/in vivo* excision assay |  |
| Leu43*sca*I_fw | caagtactctatgcggcatcagagcag | pMC477/479 | Underline indicates *Sca*I site |
| Leu43*sca*I_rev | caaagtactctggaaagcgggcagtgag | pMC477/479 | Underline indicates *Sca*I site |
