# Supplementary material for: Flipping chromosomes in deep-sea archaea
Source: PLoS Genet. 2017 Jun 19;13(6):e1006847. doi: 10.1371/journal.pgen.1006847 (PMC5495485; doi:10.1371/journal.pgen.1006847)
Supplement: S3 Table — (DOCX) [file pgen.1006847.s003.docx]

**S3 Table. Metagenomic reads mapping *(T. nautili* 66G)**

| Gene inversion | 1166 | 1333 | 1317 | 1557 |
| --- | --- | --- | --- | --- |
| original reads (position 1) | 46% | 47% | 58% | 55% |
| inversion reads (position 1) | 54% | 53% | 41% | 45% |
| original reads (position 2) | 45% | 47% | 50% | 39% |
| inversion reads (Position 2) | 54% | 53% | 49% | 60% |
|  |  |  |  |  |
|  |  |  | Average | SD |
| Reads mapped to *T. nautili* 66G chromosome excluding integrated pTN3. | | | 224.9 | 27.5 |
| Reads mapped to pTN3 | | | 310.2 | 35.6 |
| Average number of pTN3 per chromosome | | | 1.38 |  |
