# Supplementary figures and images for: Flipping chromosomes in deep-sea archaea
Source: PLoS Genet. 2017 Jun 19;13(6):e1006847. doi: 10.1371/journal.pgen.1006847 (PMC5495485; doi:10.1371/journal.pgen.1006847)

**A**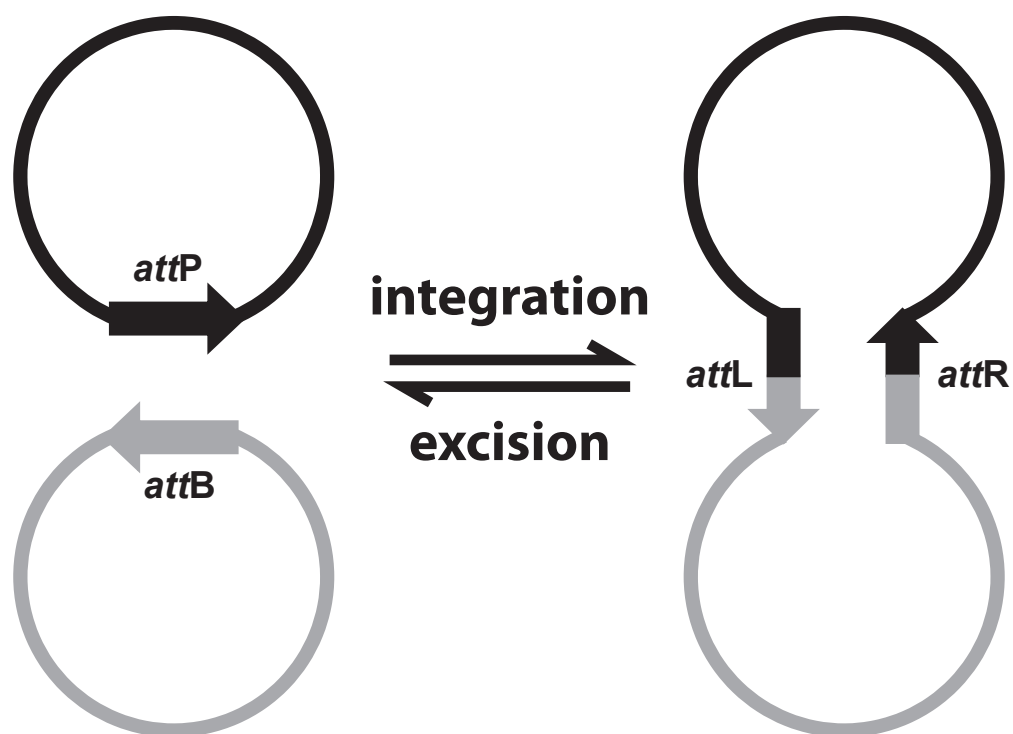**B**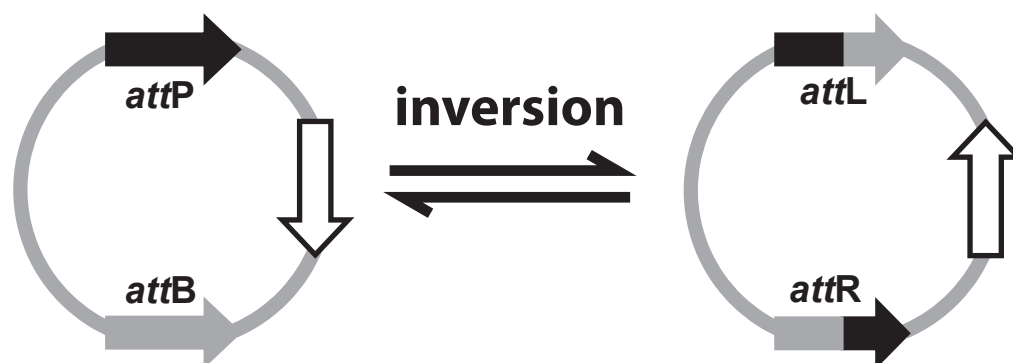

Supplement: S1 Fig — A. The intermolecular site-specific integration between cognate attP and attB sites generates a co-integrate with recombined attL and attR sites in direct orientation. The reverse reaction of excision regenerates the original components. B. In the intramolecular site-specific inversion reaction, the att sites are in opposite orientation. This reaction is reversible as well. (PDF) [file pgen.1006847.s004.pdf]

**A**

S4 Fig .

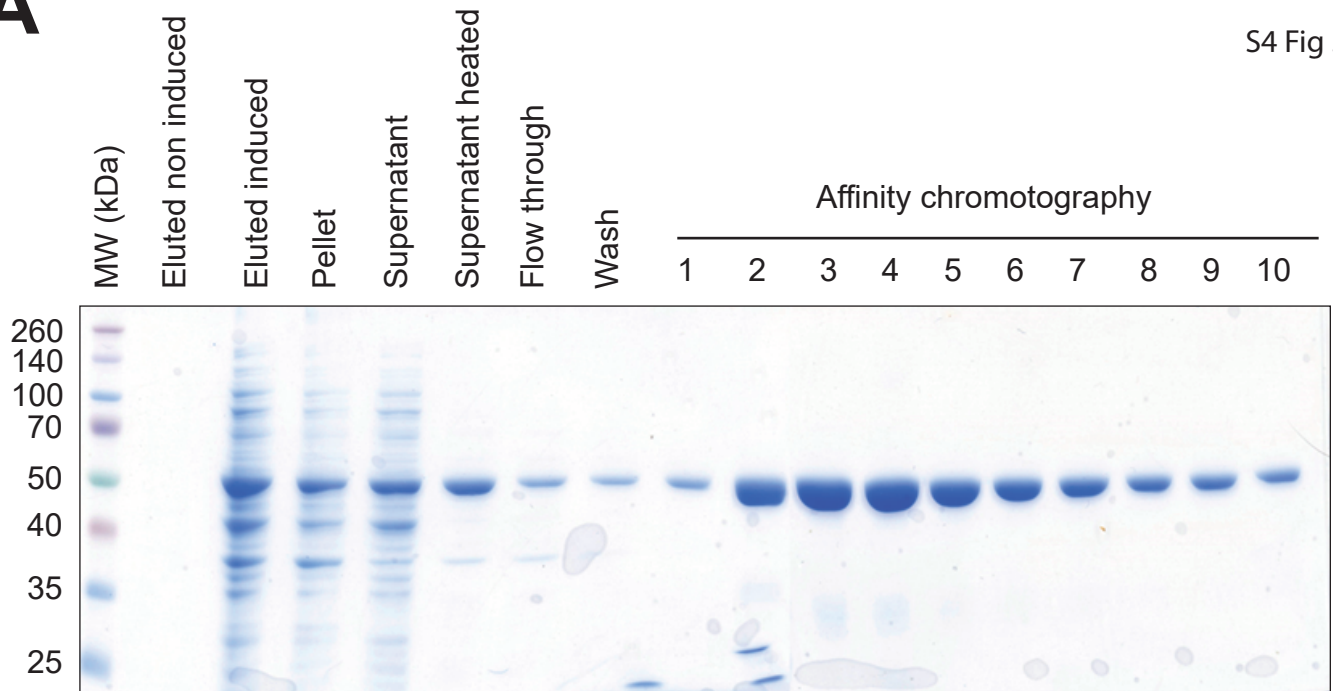**B**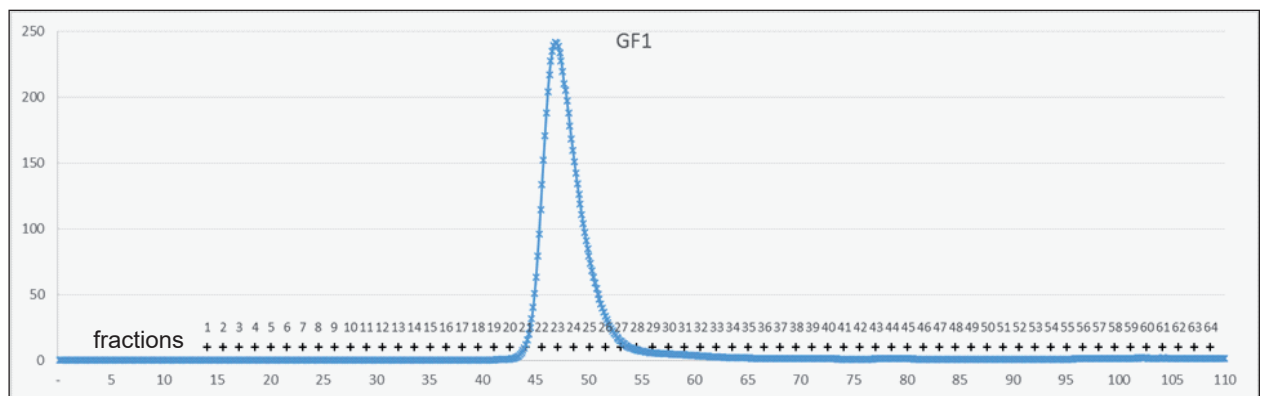**C**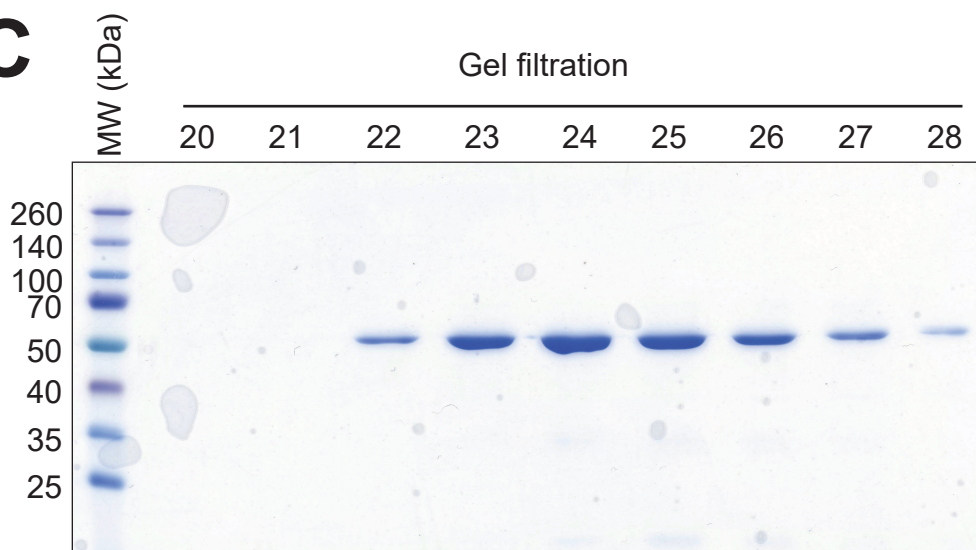

Supplement: S4 Fig — A. Protein expression was induced with 1mM IPTG in 1L of LB medium; cells harvested by centrifugation, and lysed by sonication. The soluble fraction of the sonicate was heated at 65°C for 10 minutes, and denatured proteins removed by centrifugation and by passing through a 0.45 μm filter. Strep-tagged proteins were purified by affinity fractionation using a Strep-Tactin column (IBA Lifesciences) as recommended by the supplier. B. Strep-Tactin fractions 4 and 5 were pooled and submitted to gel filtration (Superdex 200 16/600, GE Healthcare). C. Gel filtration fractions 21 to 31 were pooled and the purified protein was concentrated with an Amicon 3kDa cutoff concentrator (Millipore), aliquoted and stored at -80°C. (PDF) [file pgen.1006847.s007.pdf]

S6 Fig.

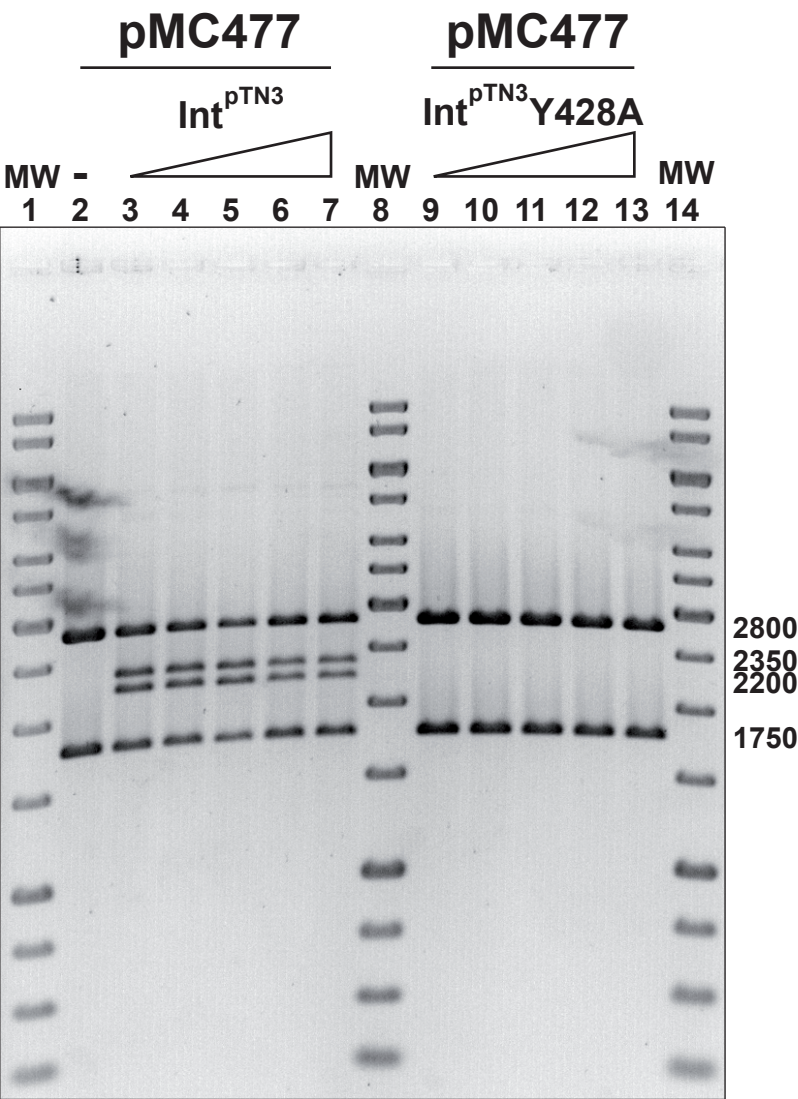

Supplement: S6 Fig — Increasing amounts of wild type IntpTN3 and mutated IntpTN3Y428A enzymes were incubated with plasmid pMC477 as substrate to analyze the inversion properties. The experimental conditions are those of the standard integrase assay (see Material and methods) except that increasing amounts of enzyme were used: 0.5, 1, 1.5, 2.5 and 5μg, respectively. No inversion is detectable with IntpTN3Y428A. (PDF) [file pgen.1006847.s009.pdf]

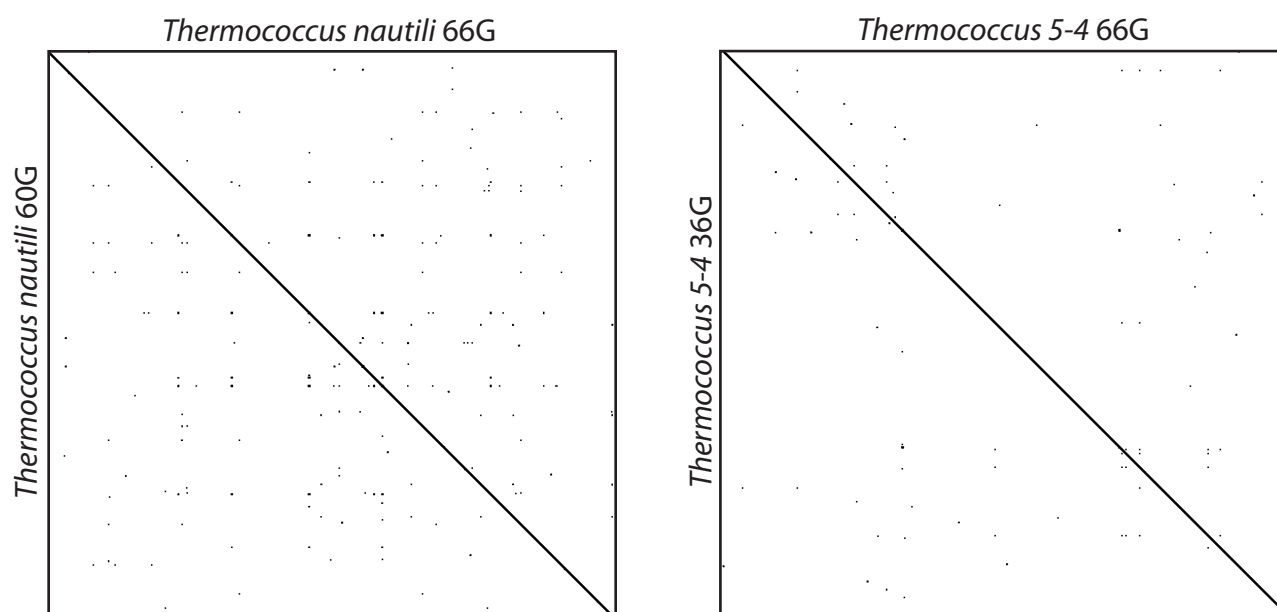

Supplement: S7 Fig — Dotplot alignment of the prominent genomes obtained after T. nautili 60G and 66G subculturing (left) and T. 5–4 36G and 66G (right). (PDF) [file pgen.1006847.s010.pdf]

A

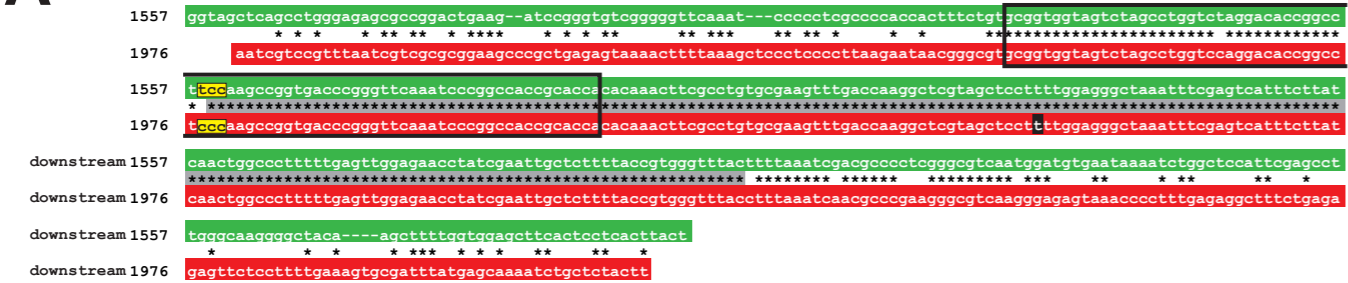

B

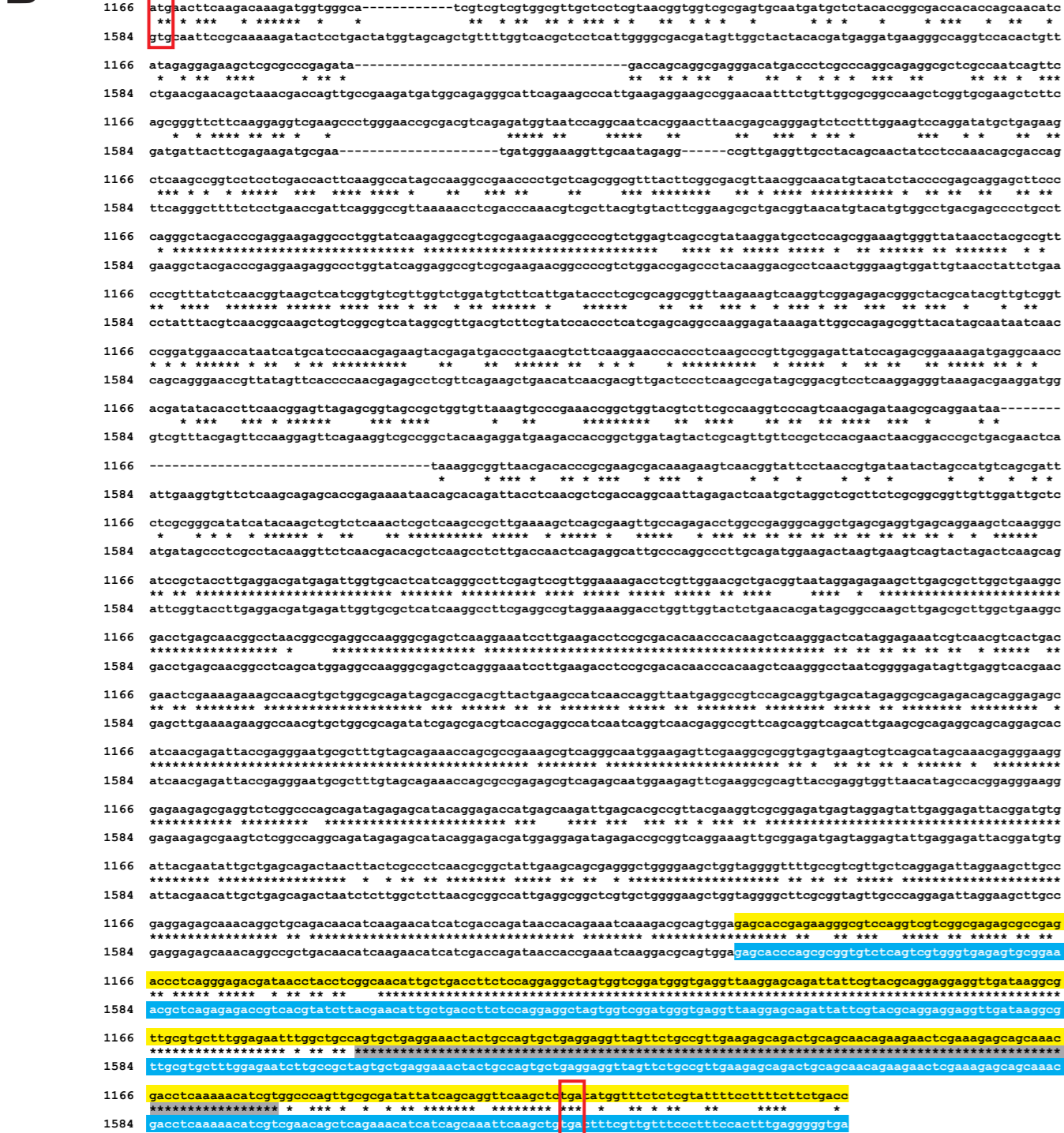

C

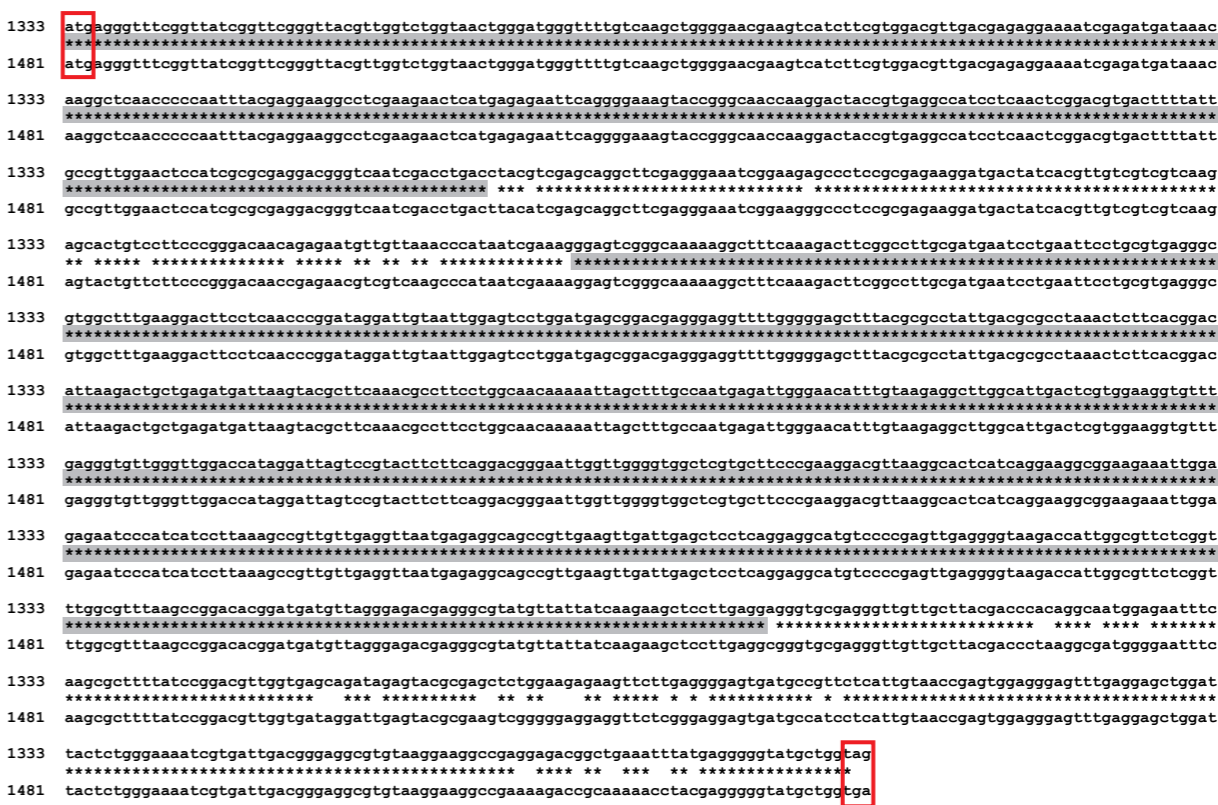

D

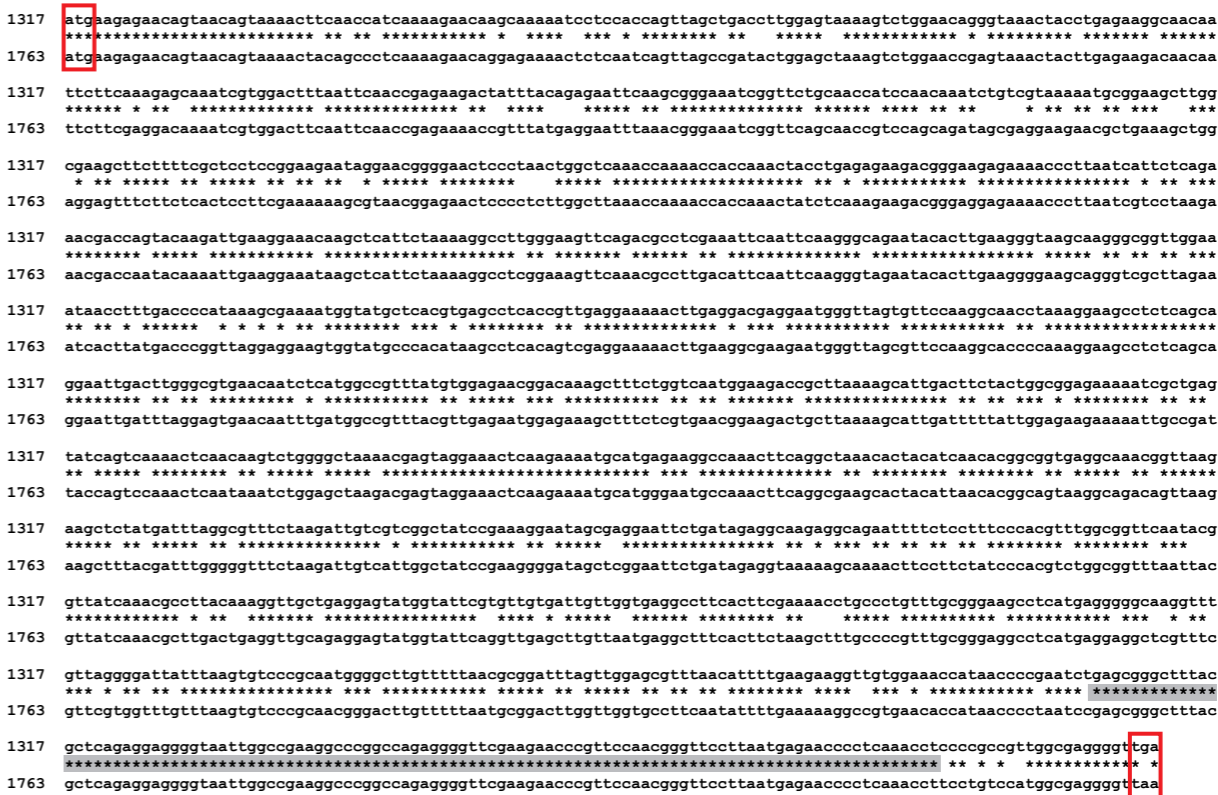

Supplement: S8 Fig — The sequences corresponding to the four genomic crossovers observed in T. nautili 60G and 66G were identified each time in pairs of paralogous genes shown aligned here. The sequences blocked in grey throughout the figure refer to perfectly conserved DNA segments in each paralogous pair where recombination occurred. Short sequences boxed in red refer to open reading frames start and stop codons when applicable (see also Fig 7 for throughout consistent color-coding). Panel A shows the alignment between segments overlapping tRNAGly genes BD01_1557 and BD01_1976. The precise regions corresponding to both tRNAGly genes are boxed in black. DNA segments cloned in pCB548 indicated by green blocks refer to BD01_1557-related sequences while red blocks correspond to BD01_1976-related sequences. The BD01-1976 nucleotide highlighted in black corrects a sequencing error in the original T. nautili genome sequence. A 176bp segment (grayed) is perfectly conserved between BD01_1557 and BD01_1976. Gly anticodons are boxed in yellow color. Panel B displays the alignment between methyl accepting chemotaxis genes BD01_1166 and BD01_1584. DNA segments cloned in pCB552 indicated by yellow blocks refer to BD01_1166-related sequences while blue blocks correspond to BD01_1594-related sequences A 176bp segment (grayed) is perfectly conserved between BD01_1166 and BD01_1584. Panel C displays the alignment between UDP-glucose-6 dehydrogenase genes BD01_1333 and BD01_1481. The two separate regions of extended sequence identity (I and II) are found between these genes respectively 284 and 620bp long (greyed). The presence of gene conversion in the interval between these two regions suggests that both were presumably involved in distinct crossover events. Panel D shows the alignment between transposase genes BD01_1317 and BD01_1763. The shortest recombination segment (104bp, grayed) is shared between these two paralogous genes. (PDF) [file pgen.1006847.s011.pdf]

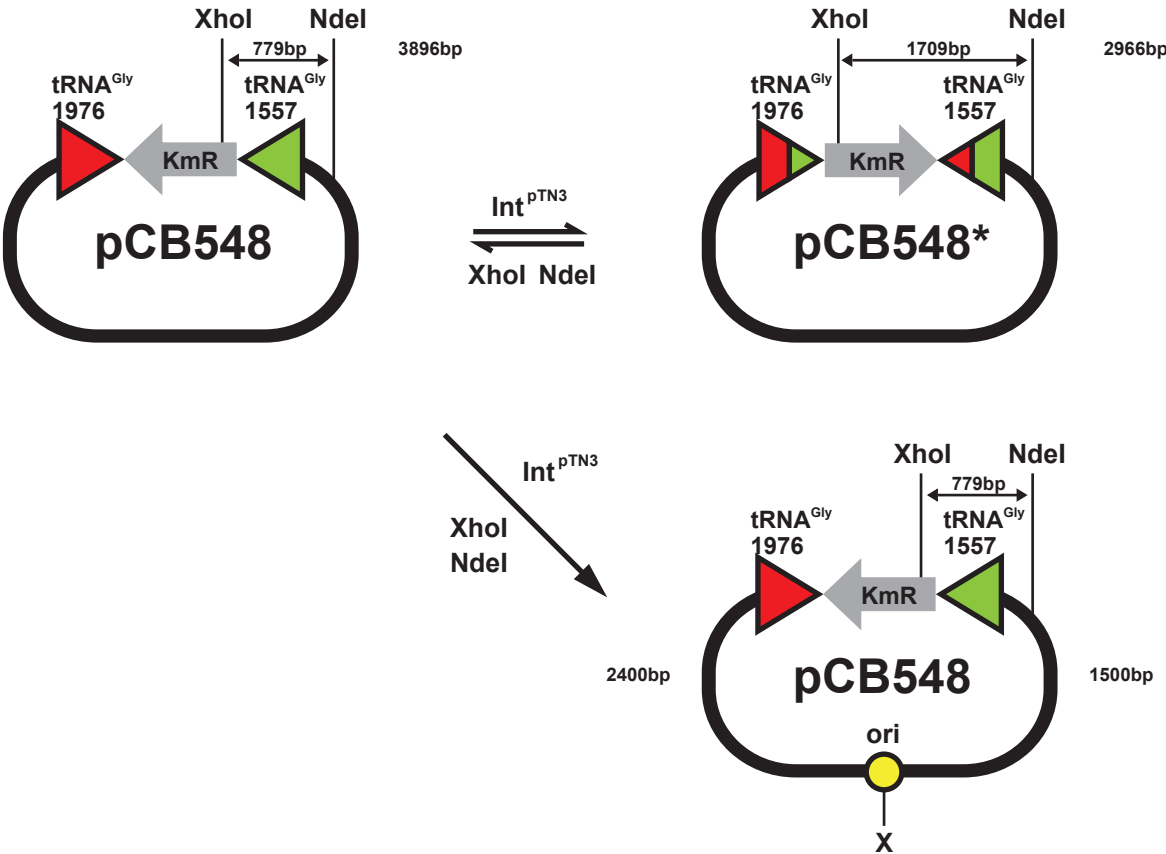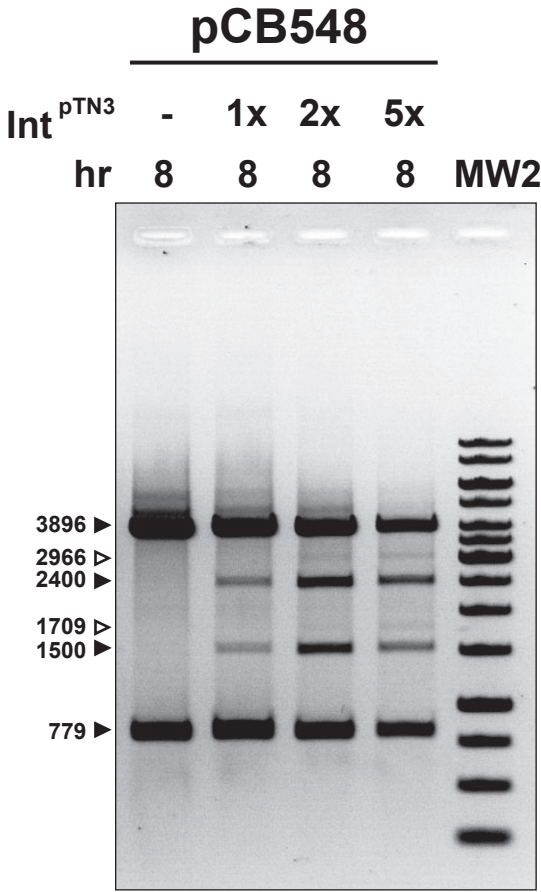

Supplement: S10 Fig — Circular plasmid pCB548 (4675bp) treated with IntpTN3 and digested with XhoI-NdeI endonucleases generates bands of 2966 and 1709bp due to integrase-promoted low sequence specificity recombination (white arrowheads). The original larger 3896bp XhoI-NdeI fragment undergoes an additional double-stranded cut at the plasmid ColE1 origin of replication to generate fragments of ~2400 and ~1500bp (black arrowheads). IntpTN3 concentration multipliers refer to the standard assay described in Materials and Methods. (PDF) [file pgen.1006847.s013.pdf]

**A**

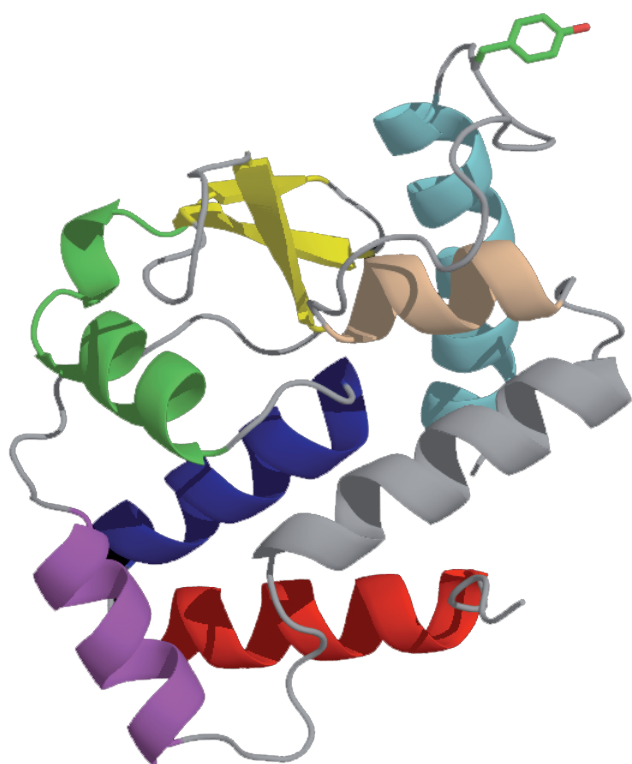

**B**

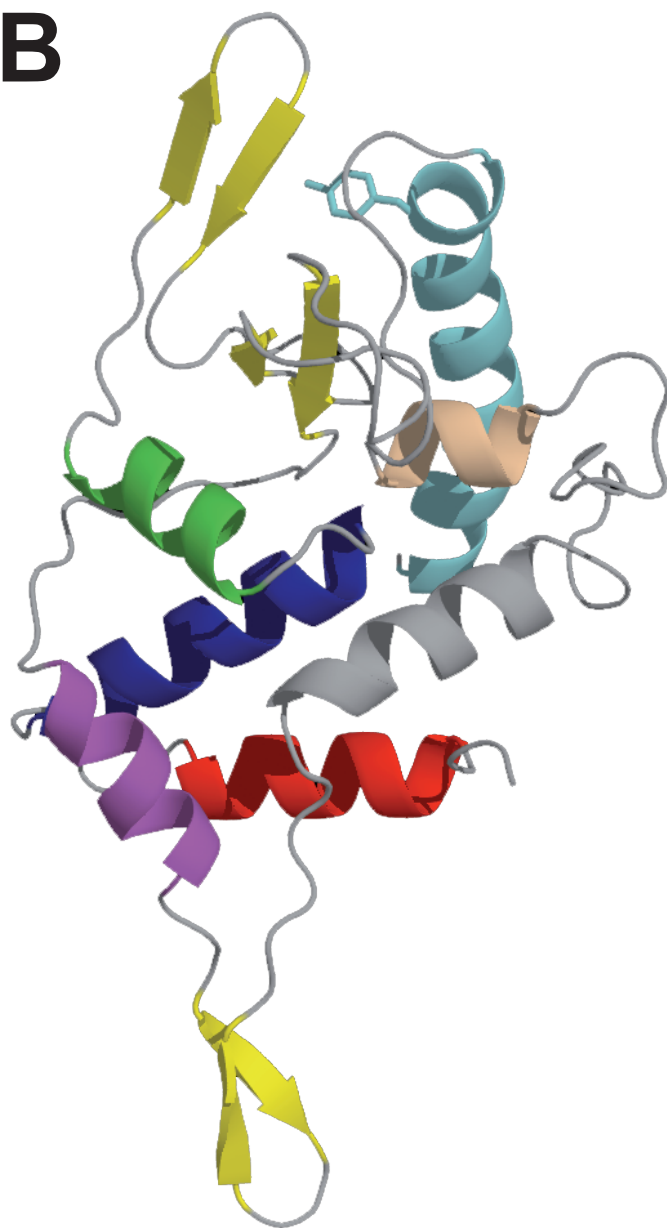

Supplement: S12 Fig — The catalytic domain of IntpTN3 (B) was modeled using Phyre2 [Reference 5 in S1 Text] and compared using PyMol (The PyMOL Molecular Graphics System, Version 1.8 Schrödinger, LLC.) with the tridimensional structure of the integrase of Sulfolobus solfataricus virus SSV1 (PDB 3VCF) (A) determined by Zhan et al [Reference 6 in S1 Text]. The IntpTN3 catalytic tyrosine residue is highlighted. (PDF) [file pgen.1006847.s015.pdf]
