# Supplementary material for: Flipping chromosomes in deep-sea archaea
Source: PLoS Genet. 2017 Jun 19;13(6):e1006847. doi: 10.1371/journal.pgen.1006847 (PMC5495485; doi:10.1371/journal.pgen.1006847)
Supplement: S2 Fig — A. The comparison between the replicative and the chromosomal integrated forms of plasmid pTN3 enabled us to reconstitute the integration event. A stretch of 41bp is shared by both attP and attB sites. The nucleotides corresponding to the leucine anticodon are underlined. Upon integration, the integrase gene is disrupted and a full length tRNALeu gene is reconstituted although separated from its original promoter. An excision event would regenerate the original recombination partners. B. DNA sequence alignment between the integrase gene of pTN3 (black) and the tRNALeu gene (red). The start and stop codons of the integrase open reading frame are boxed in blue. The integration sites attP and attB as defined by Krupovic & Bamford [45] are boxed in their respective color. (PDF) [file pgen.1006847.s005.pdf]

S2 Fig.

A

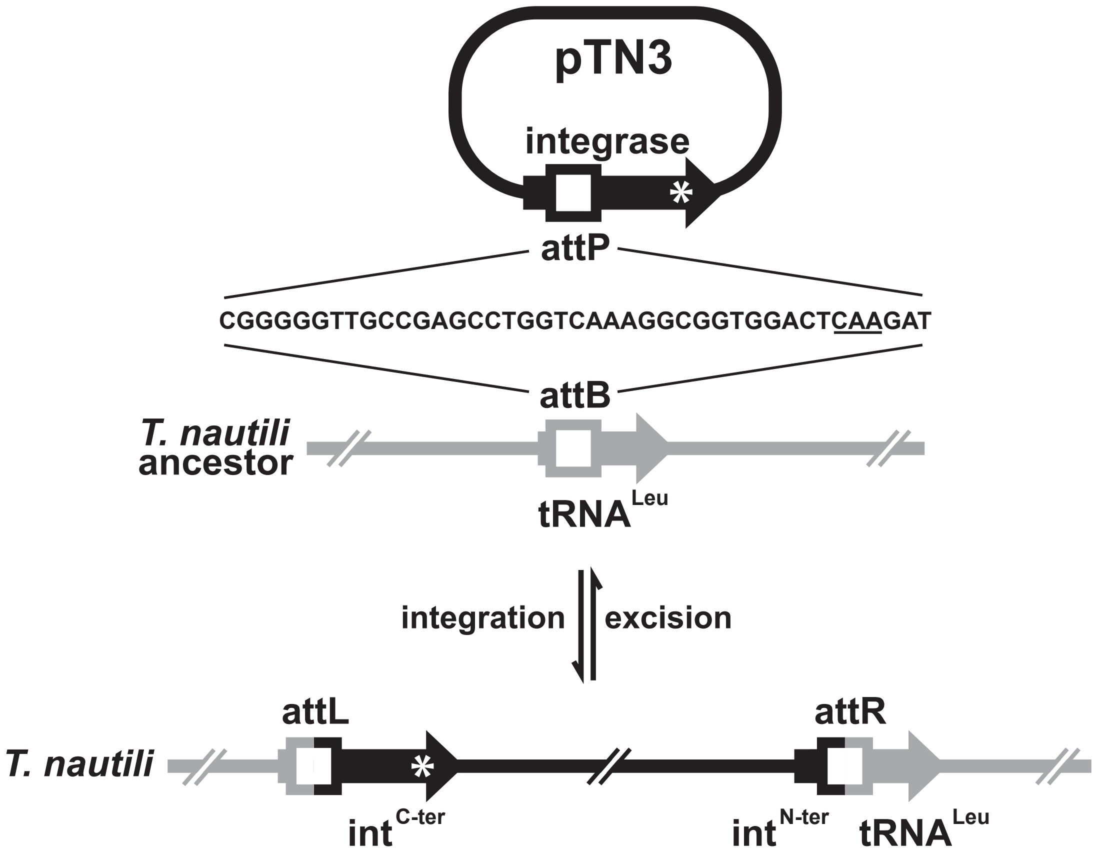

B

```
intpTN3                                                                                  ATGGTAAAATCGGGT
intpTN3   GGTGTGTACGTACACTCCCAAGCGACCGGAGAGGAGCAGGCCGGAGCGCGGAAGCGGAGGCGTCCGAGGCGCCTTTCCCCGCGTCTGT
intpTN3   ACATTACGCTACCGCCAGAAATCTATCGGAAGGCCAAGGAGCGCTGGGATAACGTGAGCCGAATCATCGCAAGCCTGCTTGAGGTGGC
intpTN3   TTTGGCTGAGGATTTAACGGTCGAGGAGGTCGTGACGGCCGTAACGCTCCTTAGGAGTGGCGCTTTGGTGGTGAATTCGCCTTCGAGC
                            attB
tRNALeu   GCGGGGGTTGCCGAGCCTGGTCAAAGGCGGTGGACTCAAGATCCACTCCCGCAGGGGTTCCGGGGTTCAAATCCCCGCCCCCGCACCA
          ****************************************** * **         *      **       *  *    *  ** **
intpTN3   GCGGGGGTTGCCGAGCCTGGTCAAAGGCGGTGGACTCAAGATGCTCTTTTTCCCCGAATGAAGGCCTCTCCCGTCAGAACGACAACA
                            attP
intpTN3   AAGAAGAGCCGAGCGCCGATAACGTTTTTACAGGAAAGGCTTTGATAGACTCAACGGCCAAAATCCACTATGGTCGTGATAGACAGAA
intpTN3   ATACATCGAATGGGTGAAACGGCGCACGCCAAGCATGGCCGACAAATACATTTCTCTGCTTGACAAGTACCTCTGGGGAAAGAAAGCC
intpTN3   AATACTCCAGAGGACCTCCGGCGCATTGTAGAAGCTATCCCTCCCACCAGGGGAGGCTTTCCCAATAGGCATGCCTACATGGCGTTGA
intpTN3   GGAGCTACATTAACTTCCTTGTGGATACCGGAAAGCTGAGGAAGAGTGAGGCCATTGACTTCAAGGCCGTGATTCCGAACGTTAAGAC
intpTN3   CAACGCTCGCGCTGAATCCGCGAAGGTCATAACGGTTGAGGACATTCGTGAGATGTTCAACCAGCTCAAGGGGAAGAACGAGACGATT
intpTN3   CTCAGAGCGCGCAAGCTTTACCTCAAGCTTCTCGCCTTTACAGGTCTCAGGGGAGACGAGGTCCGCGAGCTGATGAACCAGTTCGACC
intpTN3   CGAGGGTTATTGACGAGACATTCAAGGCCTTTGGCCTTCCTGAGGAATACAAGGAGAAGATAGCGGTCTATGATATGGAGCGGGTGAA
intpTN3   GATTAAGACGAGGAGGAGTCAGACGAAGCGTGGCTATGTCGCGGTCTTTCCCGCTGAGCTCGTTCCCGAGCTGGAGTGGTTCAGGAGC
intpTN3   ACTGGGTACAAACTCACTGCGGACAACTCTGATAAGCATAAGCTGTTCAGGGATTCCAAGGAGGTTAAGGACCTGGCCTTGCTGAGAA
intpTN3   AGTTCTGGCAGAACTTCATGAACGACAATGTGATGAGCACGGTTCCAAACCCTCCTGCTGATACCTGGCACCTCATTGAGTTCCTCCA
intpTN3   GGGACGCGCTCCCAAAAACGTGGGTGGCAGGAACTACCGCTGGAACGTCAAAAACGCCGTGAGAATCTATTATTACATGGTGGACAAA
intpTN3   TTGAAAGAGGAGCTGGGGATTCTGGAGCTTTAG
```
