# Supplementary material for: Flipping chromosomes in deep-sea archaea
Source: PLoS Genet. 2017 Jun 19;13(6):e1006847. doi: 10.1371/journal.pgen.1006847 (PMC5495485; doi:10.1371/journal.pgen.1006847)
Supplement: S3 Fig — A. Alignment of IntpTN3 with tyrosine recombinases from the three domains of life. The protein sequence of IntpTN3 (WP_022547007.1) is aligned using Praline [Reference 4 in S1 Text] with the reconstituted integrase from T. kodakarensis TKV4 and other previously characterized tyrosine recombinases from the three domains of life. These recombinases consist of the integrases from Sulfolobus Spindle Viruses SSV1 (P20214.1) and SSV2 (NP_944456.1), phage λ integrase (ALA45781.1), phage HP1 integrase (NP_043466.1), XerD resolvase from Escherichia coli (NP_417370.1) and FLP recombinase from Saccharomyces cerevisiae 2μ plasmid (P03870.1). The region corresponding to the catalytic signatures (BoxI, Kβ, BoxII) of crystallized tyrosine recombinases are boxed in light gray. The predicted residues composing IntpTN3 catalytic site are shown (R..K..AxxR..Y) and the catalytic tyrosine residue is indicated by a black arrow. The color code refers to the extent of residue conservation at each position as show in the color scale. B. Alignment of IntpTN3 with IntTKV4 and the hyperthermophilic tyrosine recombinases IntSSV1 and IntSSV2. Global protein sequence similarities were computed with the Needleman-Wunsch algorithm (Needle EMBOSS, http://www.ebi.ac.uk/Tools/psa/emboss_needle/): IntpTN3-IntTKV4: 93.6%; IntpTN3-IntSSV1: 33.0% and IntpTN3-IntSSV2: 31.2%. (PDF) [file pgen.1006847.s006.pdf]

S3 Fig.

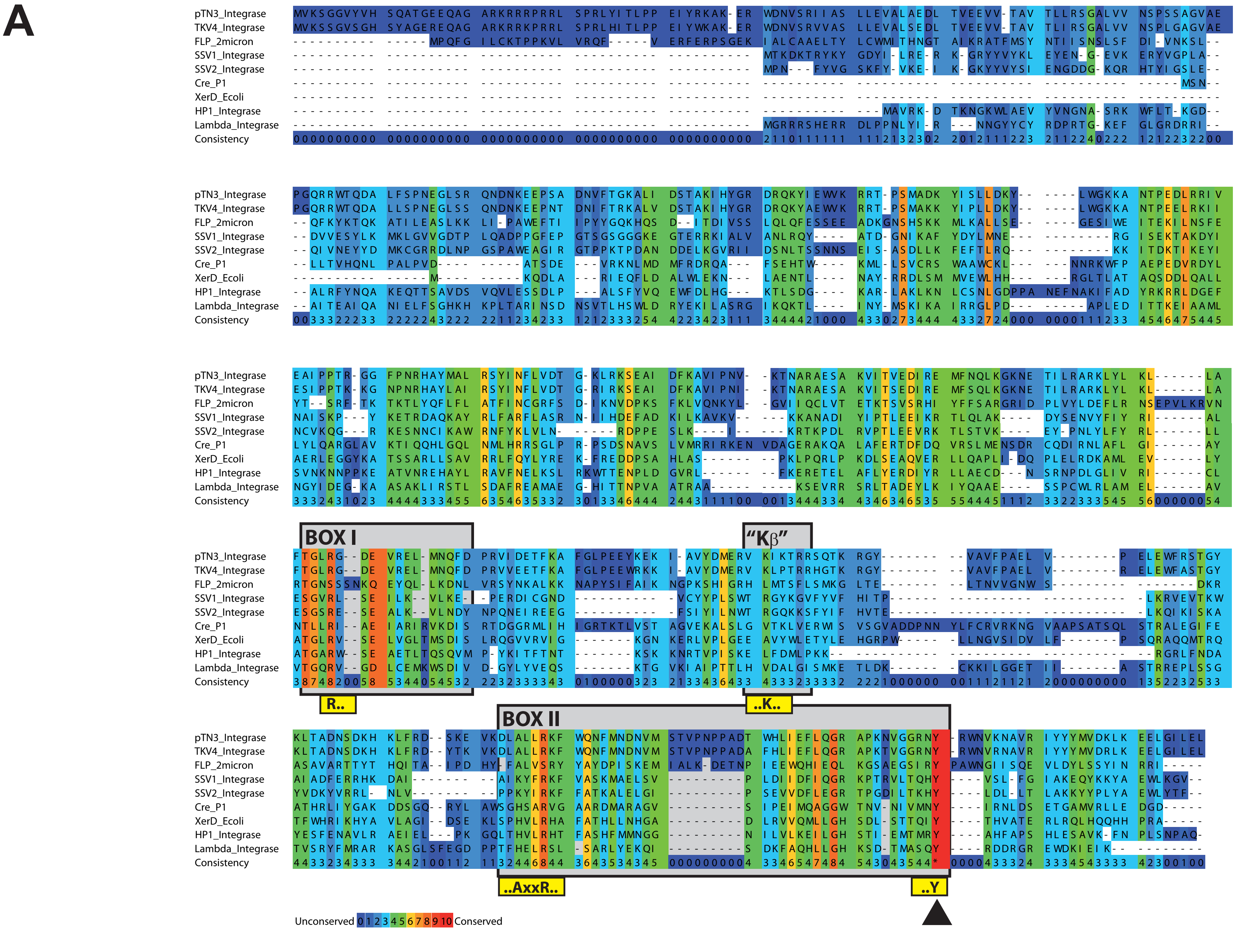
A
pTN3_Integrase
TKV4_Integrase
FLP_2micron
SSV1_Integrase
SSV2_Integrase
Cre_P1
XerD_Ecoli
HP1_Integrase
Lambda_Integrase
Consistency
BOX I
"Kβ"
R..
..K..
BOX II
..AxxR..
..Y
Unconserved 0 1 2 3 4 5 6 7 8 9 10 Conserved

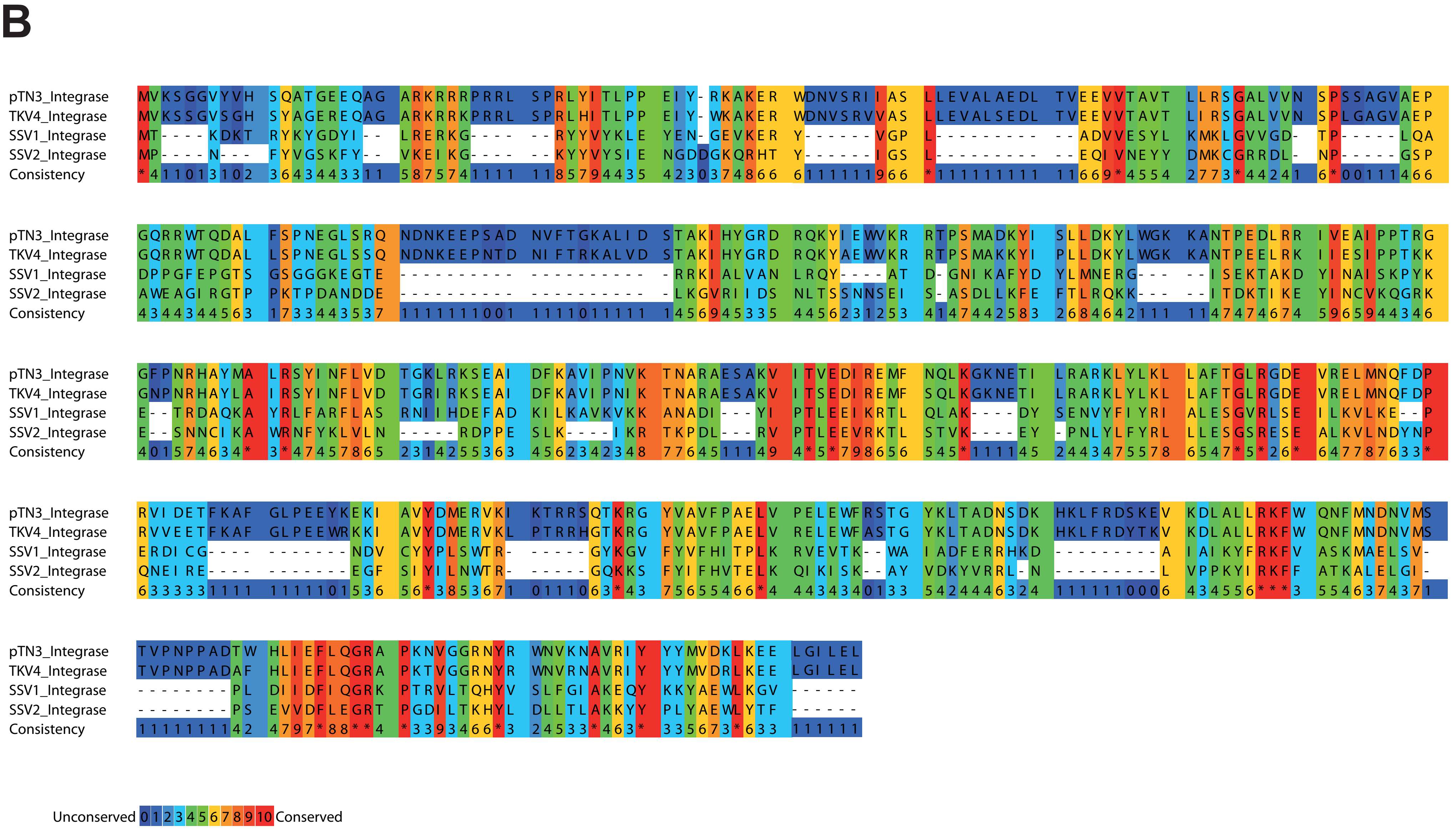
B
pTN3_Integrase
TKV4_Integrase
SSV1_Integrase
SSV2_Integrase
Consistency
Unconserved 0 1 2 3 4 5 6 7 8 9 10 Conserved
