# Supplementary material for: Flipping chromosomes in deep-sea archaea
Source: PLoS Genet. 2017 Jun 19;13(6):e1006847. doi: 10.1371/journal.pgen.1006847 (PMC5495485; doi:10.1371/journal.pgen.1006847)

A

attB deletion	Dimerisation	Sequence	Plasmid
Leu WT	ND	GCGGGGGTTGCCGAGCCTGGTCAAAGGCGGTGGACTCAAGATCCACTCCCGCAGGGGTTCCGGGGTTCAAATCCCCGCCCCCGCACCA	-
Leu 43-88	-	CCACTCCCGCAGGGGTTCCGGGGTTCAAATCCCCGCCCCCGCACCA	pJO425
Leu 2-88	+	CGGGGGTTGCCGAGCCTGGTCAAAGGCGGTGGACTCAAGATCCACTCCCGCAGGGGTTCCGGGGTTCAAATCCCCGCCCCCGCACCA	pJO322
Leu 2-82	+	CGGGGGTTGCCGAGCCTGGTCAAAGGCGGTGGACTCAAGATCCACTCCCGCAGGGGTTCCGGGGTTCAAATCCCCGCCCCC	pMC435
Leu 2-72	+	CGGGGGTTGCCGAGCCTGGTCAAAGGCGGTGGACTCAAGATCCACTCCCGCAGGGGTTCCGGGGTTCAAAT	pMC433
Leu 2-61	+	CGGGGGTTGCCGAGCCTGGTCAAAGGCGGTGGACTCAAGATCCACTCCCGCAGGGGTTCC	pMC431
Leu 2-47	+	CGGGGGTTGCCGAGCCTGGTCAAAGGCGGTGGACTCAAGATCCACT	pMC429
Leu 2-45	+	CGGGGGTTGCCGAGCCTGGTCAAAGGCGGTGGACTCAAGATCCA	pMC443
Leu 2-44	+	CGGGGGTTGCCGAGCCTGGTCAAAGGCGGTGGACTCAAGATCC	pMC449
Leu 2-43	-	CGGGGGTTGCCGAGCCTGGTCAAAGGCGGTGGACTCAAGATC	pMC441
Leu 2-42-*attP*	-	CGGGGGTTGCCGAGCCTGGTCAAAGGCGGTGGACTCAAGAT	pJO421
Leu 5-44	-	GGGTTGCCGAGCCTGGTCAAAGGCGGTGGACTCAAGATCC	pJO459
Leu 8-44	-	TTGCCGAGCCTGGTCAAAGGCGGTGGACTCAAGATCC	pJO461
Leu 12-44	-	CGAGCCTGGTCAAAGGCGGTGGACTCAAGATCC	pJO463
Leu 17-44	-	CTGGTCAAAGGCGGTGGACTCAAGATCC	pJO465
		***	
attP		CGGGGGTTGCCGAGCCTGGTCAAAGGCGGTGGACTCAAGAT	

B

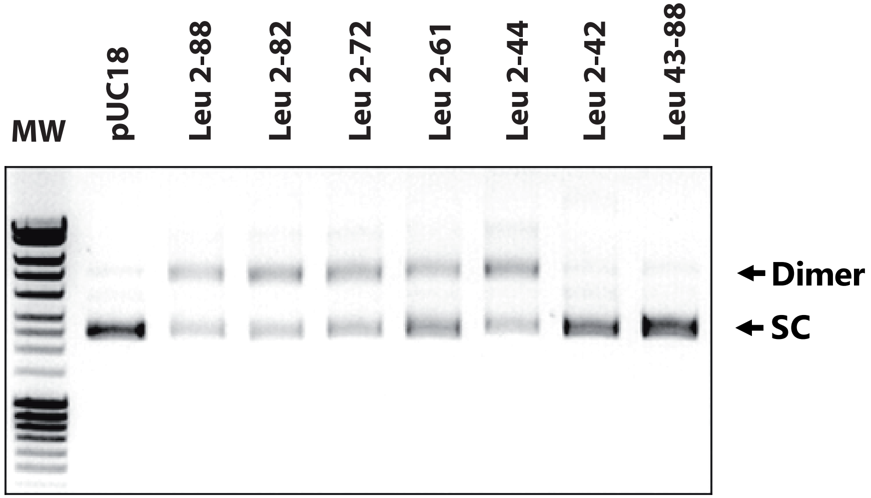

Supplement: S5 Fig — The Integrase dimerization test was used to determine the minimal site required for IntpTN3 tRNALeu × tRNALeu recombination on nested deletions carried by plasmid templates. A. DNA sequence of the nested deletions. DNA segments corresponding to theses sequences were annealed and cloned directionally in pUC18. B. The resulting supercoiled plasmids were incubated with purified IntpTN3 in a standard reaction and scored for dimer formation by agarose gel electrophoresis where only relevant reactions are shown. The dimerization-proficient sequences in Panel A are marked as positive. It is noteworthy that the Leu41 site, a site corresponding to the 41bp of sequence identity shared by both attP and attB is not a sufficient substrate for this reaction. Therefore, the minimal site for efficient dimerization is Leu2-44 with a size of 43bp. The asterisks indicate the extent of sequence identity between chromosomal attB and pTN3 attP. The leucine CAA anticodon is underlined. (PDF) [file pgen.1006847.s008.pdf]
