# Supplementary material for: Flipping chromosomes in deep-sea archaea
Source: PLoS Genet. 2017 Jun 19;13(6):e1006847. doi: 10.1371/journal.pgen.1006847 (PMC5495485; doi:10.1371/journal.pgen.1006847)
Supplement: S9 Fig — Specific sequences surrounding tRNA gene BD01_1976 are blocked in red while specific sequences surrounding tRNA gene BD01_1557 are blocked in green. Relevant anticodon sequences are boxed in yellow color. Two nucleotide mismatches between these tRNA genes are blocked in black. The tripartite composition of these DNA segments is further highlighted by blocking in grey color the stretch of identical sequenced shared by the DNA fragments carrying BD01_1976 and BD01_1557. Panel A depicts the sequence of steps involved in generating a suitable recombinant fragment for DNA sequencing. Plasmid pCB548 carries DNA segments containing T. nautili tRNAGly-encoding genes BD01_1976 and BD01_1557 in inverted orientation and separated by a Kanamycin resistance determinant originating from pUC4K. The exact sequence of the cloned DNA segments encompassing BD01_1976 & BD01_1557 is displayed in S8A Fig. The inversion reaction was performed as shown in Fig 7B: an EcoRI-ScaI fragment originating from pCB548 was incubated with IntpTN3 after which the 601bp EcoRI-NruI fragment generated by IntpTN3 recombination was gel-purified, PCR-amplified with the forward primer 5’-ccgtttaatcgtcgcgcggaagc-3’ targeting the upstream sequence of the tRNAGly gene BD01_1976 and the reverse primer 5’-cccgttgaatatggctcataacaccc-3’ targeting the beginning of the KanR cassette. The resulting fragment was submitted to Sanger DNA sequencing using the forward primer mentioned above. Panels B and C display also the alignment between the 5’ half of both tRNA genes and the minimal Leu2-44 segment involved in IntpTN3 site-specific recombination. Panel D shows the result of the DNA sequencing reaction. The crossover point in the recombination reaction occurred precisely downstream of the two nucleotide mismatches mentioned above, in the sequence blocked in grey corresponding to the 3’ half of the tRNA genes and strictly conserved sequences immediately following. The sequences boxed in black in Panels B,C and D correspo [file pgen.1006847.s012.pdf]

S9 Fig.

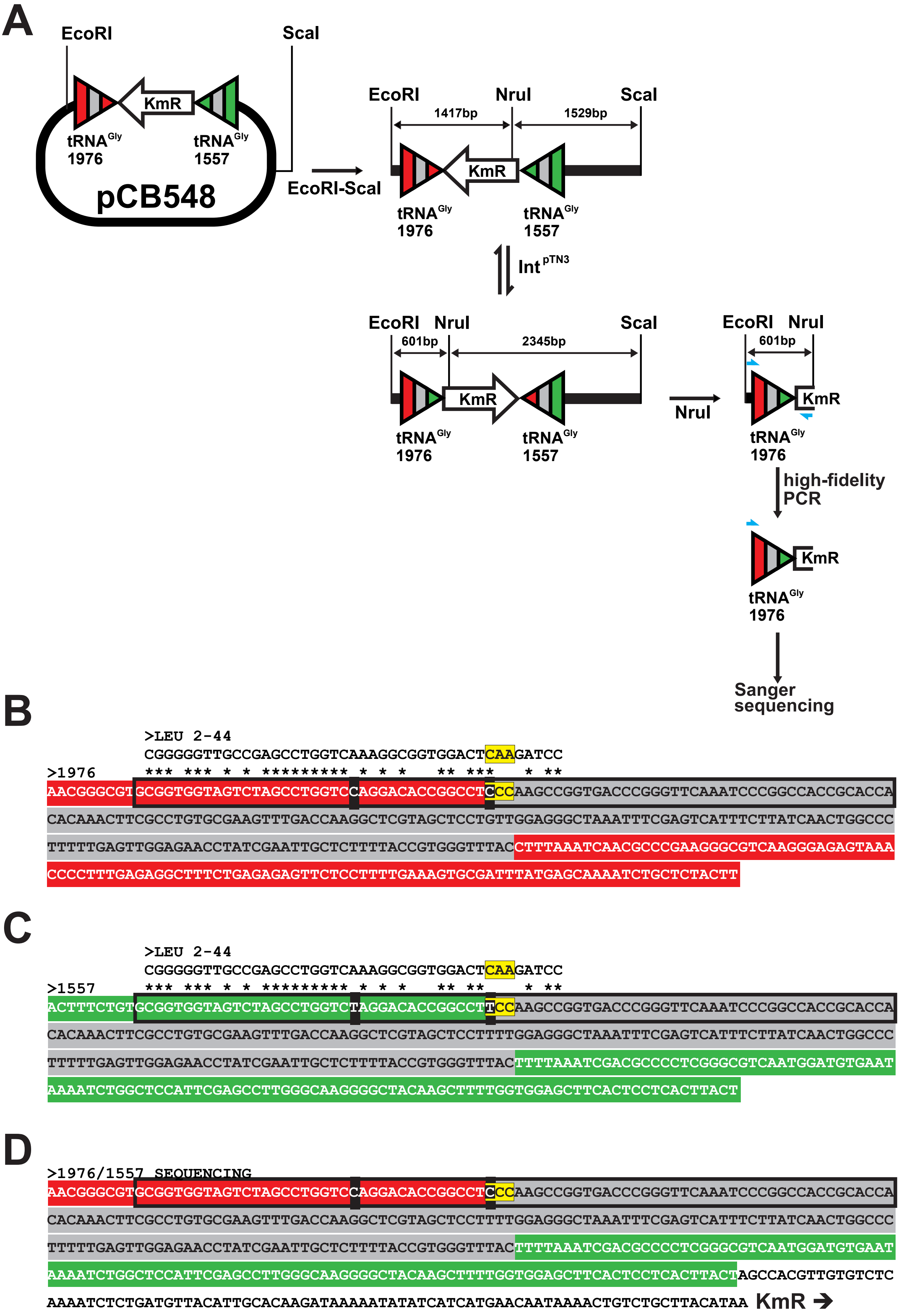
A
EcoRI
ScaI
KmR
tRNA Gly 1976
tRNA Gly 1557
pCB548
EcoRI-ScaI
EcoRI
NruI
ScaI
1417bp
1529bp
KmR
tRNA Gly 1976
tRNA Gly 1557
Int pTN3
EcoRI NruI
ScaI
601bp
2345bp
KmR
tRNA Gly 1976
tRNA Gly 1557
NruI
EcoRI NruI
601bp
KmR
tRNA Gly 1976
high-fidelity PCR
KmR
tRNA Gly 1976
Sanger sequencing
B
>LEU 2-44
CGGGGGTTGCCGAGCCTGGTCAAAGGCGGTGGACTCAAGATCC
*** *** * * ********* * * * ** *** * **
>1976
AACGGGCGTGCGGTGGTAGTCTAGCCTGGTCCAGGACACCGGCCTCCCAAGCCGGTGACCCGGGTTCAAATCCCGGCCACCGCACCA
CACAAACTTCGCCTGTGCGAAGTTTGACCAAGGCTCGTAGCTCCTGTTGGAGGGCTAAATTTCGAGTCATTTCTTATCAACTGGCCC
TTTTTGAGTTGGAGAACCTATCGAATTGCTCTTTTACCGTGGGTTTACCTTTAAATCAACGCCCGAAGGGCGTCAAGGGAGAGTAAA
CCCCTTTGAGAGGCTTTCTGAGAGAGTTCTCCTTTTGAAAGTGCGATTTATGAGCAAAATCTGCTCTACTT
C
>LEU 2-44
CGGGGGTTGCCGAGCCTGGTCAAAGGCGGTGGACTCAAGATCC
*** *** * * ********* * * * ** ** * **
>1557
ACTTTCTGTGCGGTGGTAGTCTAGCCTGGTCTAGGACACCGGCCTTCCAAGCCGGTGACCCGGGTTCAAATCCCGGCCACCGCACCA
CACAAACTTCGCCTGTGCGAAGTTTGACCAAGGCTCGTAGCTCCTTTTGGAGGGCTAAATTTCGAGTCATTTCTTATCAACTGGCCC
TTTTTGAGTTGGAGAACCTATCGAATTGCTCTTTTACCGTGGGTTTACTTTTAAATCGACGCCCCTCGGGCGTCAATGGATGTGAAT
AAAATCTGGCTCCATTCGAGCCTTGGGCAAGGGGCTACAAGCTTTTGGTGGAGCTTCACTCCTCACTTACT
D
>1976/1557 SEQUENCING
AACGGGCGTGCGGTGGTAGTCTAGCCTGGTCCAGGACACCGGCCTCCCAAGCCGGTGACCCGGGTTCAAATCCCGGCCACCGCACCA
CACAAACTTCGCCTGTGCGAAGTTTGACCAAGGCTCGTAGCTCCTTTTGGAGGGCTAAATTTCGAGTCATTTCTTATCAACTGGCCC
TTTTTGAGTTGGAGAACCTATCGAATTGCTCTTTTACCGTGGGTTTACTTTTAAATCGACGCCCCTCGGGCGTCAATGGATGTGAAT
AAAATCTGGCTCCATTCGAGCCTTGGGCAAGGGGCTACAAGCTTTTGGTGGAGCTTCACTCCTCACTTACTAGCCACGTTGTGTCTC
AAAATCTCTGATGTTACATTGCACAAGATAAAAATATATCATCATGAACAATAAAACTGTCTGCTTACATAA KmR
