# Supplementary material for: Flipping chromosomes in deep-sea archaea
Source: PLoS Genet. 2017 Jun 19;13(6):e1006847. doi: 10.1371/journal.pgen.1006847 (PMC5495485; doi:10.1371/journal.pgen.1006847)
Supplement: S11 Fig — DNA sequence of the lacZ gene segments cloned in plasmids pCB538 (lac100), pCB572 (lac175) and pCB574 (lac250) (Fig 8B). (PDF) [file pgen.1006847.s014.pdf]

```
>lac100
CACTGGCCGTCGTTTTACAACGTCGTGACTGGGAAAACCCTGGCGTTACC
CAACTTAATCGCCTTGCAGCACATCCCCCTTTCGCCAGCTGGCGTAATAG

>lac175
CACTGGCCGTCGTTTTACAACGTCGTGACTGGGAAAACCCTGGCGTTACC
CAACTTAATCGCCTTGCAGCACATCCCCCTTTCGCCAGCTGGCGTAATAG
CGAAGAGGCCCGCACCGATCGCCCTTCCCAACAGTTGCGCAGCCTGAATG
GCGAATGGCGCCTGATGCGGTATTT

>lac250
CACTGGCCGTCGTTTTACAACGTCGTGACTGGGAAAACCCTGGCGTTACC
CAACTTAATCGCCTTGCAGCACATCCCCCTTTCGCCAGCTGGCGTAATAG
CGAAGAGGCCCGCACCGATCGCCCTTCCCAACAGTTGCGCAGCCTGAATG
GCGAATGGCGCCTGATGCGGTATTTTCTCCTTACGCATCTGTGCGGTATT
TCACACCGCATATGGTGCACTCTCAGTACAATCTGCTCTGATGCCGCATA
```
