# Supplementary material for: Flipping chromosomes in deep-sea archaea
Source: PLoS Genet. 2017 Jun 19;13(6):e1006847. doi: 10.1371/journal.pgen.1006847 (PMC5495485; doi:10.1371/journal.pgen.1006847)
Supplement: S1 Text — (DOCX) [file pgen.1006847.s020.docx]

**S1 Text. Supporting information references**

1. Norrander J, Kempe T, Messing J (1983) Construction of Improved M13-Vectors Using Oligodeoxynucleotide-Directed Mutagenesis. Gene 26: 101-106.

2. Bolivar F, Rodriguez RL, Greene PJ, Betlach MC, Heyneker HL, et al. (1977) Construction and Characterization of New Cloning Vehicles .2. Multipurpose Cloning System. Gene 2: 95-113.

3. Taylor LA, Rose RE (1988) A Correction in the Nucleotide-Sequence of the Tn903 Kanamycin Resistance Determinant in Puc4k. Nucleic Acids Research 16: 358-358.

4. Bawono P, Heringa J (2014) PRALINE: A Versatile Multiple Sequence Alignment Toolkit. Multiple Sequence Alignment Methods 1079: 245-262.

5. Kelley LA, Mezulis S, Yates CM, Wass MN, Sternberg MJE (2015) The Phyre2 web portal for protein modeling, prediction and analysis. Nat Protocols 10: 845-858.

6. Zhan ZY, Ouyang SY, Liang WG, Zhang ZF, Liu ZJ, et al. (2012) Structural and functional characterization of the C-terminal catalytic domain of SSV1 integrase. Acta Crystallographica Section D-Biological Crystallography 68: 659-670.
